# Age-related enhancement of the association between episodic memory and gray matter volume in medial temporal and frontal lobes

**DOI:** 10.1186/s12993-024-00237-y

**Published:** 2024-05-03

**Authors:** Shaokun Zhao, Feng Sang, Chen Liu, Fei Wang, Jiawen Liu, Chuansheng Chen, Jun Wang, Xin Li, Zhanjun Zhang

**Affiliations:** 1 State Key Laboratory of Cognitive Neuroscience and Learning, Beijing Normal University, Beijing, 100875 China; 2 BABRI Centre, Beijing Normal University, Beijing, 100875 China; 3 Department of Psychological Science, University of California, Irvine, CA 92697 USA

**Keywords:** Episodic memory, Gray matter volume, Older adults

## Abstract

**Background:**

Episodic memory (EM) deteriorates as a result of normal aging as well as Alzheimer’s disease. The neural underpinnings of such age-related memory impairments in older individuals are not well-understood. Although previous research has unveiled the association between gray matter volume (GMV) and EM in the elderly population, such findings exhibit variances across distinct age cohorts. Consequently, an investigation into the dynamic evolution of this relationship with advancing age is imperative.

**Result:**

The present study utilized a sliding window approach to examine how the correlation between EM and GMV varied with age in a cross-sectional sample of 926 Chinese older adults. We found that both verbal EM (VEM) and spatial EM (SEM) exhibited positive correlations with GMV in extensive areas primarily in the temporal and frontal lobes and that these correlations typically became stronger with older age. Moreover, there were variations in the strength of the correlation between EM and GMV with age, which differed based on sex and the specific type of EM. Specifically, the association between VEM and GMVs in the insula and parietal regions became stronger with age for females but not for males, whereas the association between SEM and GMVs in the parietal and occipital regions became stronger for males but not for females. At the brain system level, there is a significant age-related increase in the correlations between both types of EM and the GMV of both the anterior temporal (AT) system and the posterior medial (PM) system in male group. In females, both types of EM show stronger age-related correlations with the GMV of the AT system compared to males.

**Conclusions:**

Our study revealed a significant positive correlation between GMV in most regions associated with EM and age, particularly in the frontal and temporal lobes. This discovery offers new insights into the connection between brain structure and the diminishing episodic memory function among older individuals.

**Supplementary Information:**

The online version contains supplementary material available at 10.1186/s12993-024-00237-y.

## Background

Aging is becoming an increasingly pressing issue, with estimates suggesting that by 2050, 16% of the world’s population will be over 60 years old [1]. With age comes a decline in cognitive functions such as episodic memory (EM) [2, 3], which can significantly impact the quality of life of older individuals. It is, therefore, of paramount importance to understand the biological basis of EM by investigating the association between EM and brain aging and to use such evidence for the early detection of neurodegenerative diseases.

EM involves a wide range of brain regions. The most consistent findings indicate a positive correlation between EM and the gray matter volume (GMV) or the thickness of gray matter in the medial temporal lobe (MTL) [4–8]. The age-related decline in EM has been associated with decreased GMV in specific regions of MTL, including the hippocampus [5, 9], especially the right hippocampus [10, 11]; the middle and inferior temporal cortex [12]; entorhinal cortex [7]; and amygdala [12, 13]. In addition to MTL, other implicated regions include left superior temporal cortex [10] and lateral prefrontal cortex (lPFC) [14].

Three factors have been found to moderate the relation between EM and the brain: type of EM, age, and sex. First, two types of EM have been commonly studied: verbal (VEM) and spatial (SEM). They have been found to be associated with different brain regions. Suri, Topiwala [15] found that VEM was correlated with functional connectivity between temporal and frontal nodes, whereas SEM was correlated with functional connectivity between hippocampal and parietal regions. The differential neural bases for VEM and SEM may be linked to two neural systems—the anterior temporal (AT) system and the posterior medial (PM) system—that interact with the hippocampus to process different types of information [16]. The AT system is essential for familiarity recognition, emotional processing, social cognition, and semantic representation, whereas the PM system plays a crucial role in context memory retrieval and spatial navigation. Consequently, both systems are expected to be involved in EM, with the AT system being more relevant to VEM and the PM system being more relevant to SEM. However, there is limited empirical research exploring the relationship between these two types of EM and their corresponding neural systems in older adults.

Second, as mentioned earlier, EM declines with age, so it is likely that the neural bases of EM also vary by age. Some longitudinal studies have revealed that a higher rate of EM decline is associated with greater volume loss in the MTL [17, 18] and occipital lobes [12], and with the annual atrophy rate of the hippocampus [6, 9, 10]. These studies have focused on the covariation of age-related EM and brain structural changes, but they have not investigated whether this relationship varies across different age groups. However, one longitudinal study [6] has focused on the role of age in the association between GMV of the hippocampus and EM, and found that the association was significant in older adults (aged 65–80), but not in middle-aged adults (aged 55–60). Results from cross-sectional studies have also revealed similar effects. One study found that maintaining memory function throughout the adult lifespan may mitigate the typical age-related volume loss in the hippocampus. Furthermore, an age-by-EM interaction term was found to better predict GMV, suggesting that the predictive effects of EM on GMV are modulated by age across different age groups [19]. The broader literature on the association between gray matter structure and cognition has shown the pattern that the association is stronger in older adults than in younger individuals [20].More research is needed to confirm and understand the role of age in the association between EM and GMV.

Finally, sex is an important factor in EM and its neural basis. Some studies have identified differences in GMV between sexes, which can serve as a foundational basis for explaining cognitive disparities [21–24]. Generally, females tend to perform better than do males on verbal tasks, whereas males perform better than do females on visual-spatial tasks [25–27], Several studies have provided limited neurobiological underpinnings for these differences [28–30]. This difference may be due to variations in the underlying grey matter structures of the male and female brains. Specifically, compared to females, males tend to have larger volumes in the medial frontal cortex, left inferior parietal cortex (IPC), amygdala, and hypothalamus, as well as higher synaptic density in the neocortex of the temporal lobe [31]. In contrast, females have larger volumes in the frontal and temporal lobes [31] and the Broca’s (in the dorsolateral prefrontal cortex) and Wernicke’s (in the superior temporal cortex) areas [32]. Male advantage in left inferior parietal cortex (IPC), an area related to spatial relationship understanding, may explain their superior spatial information processing abilities [33], including SEM. Female advantage in the language areas (Broca’s and Wernicke’s areas) may explain their better language abilities, including VEM. Moreover, given the importance of both age and sex in EM and brain aging, it is important to consider these two factors together. Indeed, previous studies have also found that compared to females, males have a higher rate of volume loss in certain brain regions that are important for EM, including the hippocampus, amygdala [34–36], and frontal lobe [35]. Furthermore, research has shown that there is a significant interaction between age and sex in the annual percentage change of gray matter ratio (GMR) [37].

To investigate the correlation between EM and GMV variations associated with age among older individuals. we used cross-sectional data from adults aged 55–90 years from the Beijing Aging Brain Rejuvenation Initiative (BABRI). Our study first identified brain regions whose GMV showed high correlations with EM in the whole sample (and for males and females separately) and then used the sliding window approach to examine the age-related association between GMV and EM in these brain regions. The sliding window approach’s advantage is to allow us to analyze cross-sectional data to yield a developmentally continuous picture [38]. We specifically tested the following hypotheses. First, the two types of EM would have some shared gray matter structural bases, such as the Hippocampus MTL and lPFC, but also distinct neural bases, such as VEM being associated with Broca’s and Wernicke’s areas and SEM being associated with the IPC. Secondly, the relationship between EM and GMV is correlated with age, and supports the existence of sex differences in gray matter structure for various types of EM.

## Results

### Descriptive statistics and sex differences in demographic characteristics, total intracranial volume, and EM performance

As shown in Table 1, compared to females, males had significantly more years of education (t = 2.71, *p* = 0.007), larger total intracranial volume (TIV) (t = 25.01, *p* < 0.001), higher SEM scores (t = 3.69, *p* < 0.001), but lower VEM scores (t=-4.40, *p* < 0.001). There was no significant sex difference in age (t = 0.27, *p* = 0.785).

**Table 1:** Descriptive statistics and sex differences

	Mean ± SD			t (*p*)
	All participants (*N* = 926)	Males (*N* = 463)	Females (*N* = 463)	
Age	68.09 ± 6.96	68.15 ± 6.88	68.03 ± 7.05	0.27 (0.785)
Education	11.75 ± 3.28	12.04 ± 3.29	11.46 ± 3.24	2.71 (0.007)
TIV	1407.33 ± 140.03	1496.25 ± 116.86	1318.41 ± 98.77	25.01 (< 0.001)
VEM	27.44 ± 9.62	26.04 ± 9.21	28.82 ± 9.83	-4.40 (< 0.001)
SEM	13.66 ± 6.99	14.50 ± 6.86	12.82 ± 7.03	3.69 (< 0.001)

*Note* TIV: total intracranial volume; VEM: verbal episodic memory; SEM: spatial episodic memory

### Differences in VEM and SEM between age groups

Multiple linear regression models were used to examine the relationship between age and EM after controlling for years of education (as well as sex in the analysis of the total sample). Results showed that both VEM and SEM were negatively correlated significantly with age for the total sample (VEM: β=-0.04, t=-8.72, df = 922, *p* < 0.001; and SEM: β=-0.03, t=-6.56, df = 922, *p* < 0.001), for males (VEM: β=-0.04, t=-5.80, df = 460, *p* < 0.001; and SEM: (β=-0.02, t=-3.44, df=,460 *p* < 0.001), and for females (VEM: β=-0.04, t=-6.34, df = 460, *p* < 0.001; and SEM: β=-0.04, t=-5.62, df = 460, *p* < 0.001). Direct comparisons of linear slopes of memory as a function of age between males and females via the inclusion of interaction terms (sex×age) showed no significant sex difference in age effect for either SEM or VEM (interaction terms *p* > 0.05).s.

### The relationship between GMV and two types of EM

Multiple linear regression model was utilized to investigate the relationship between GMV and EM performance, with results corrected for false discovery rates (FDR = 0.05). The covariates included age, years of education, and TIV (sex was used solely for the analysis of the entire sample).

#### VEM

**Fig. 1:**
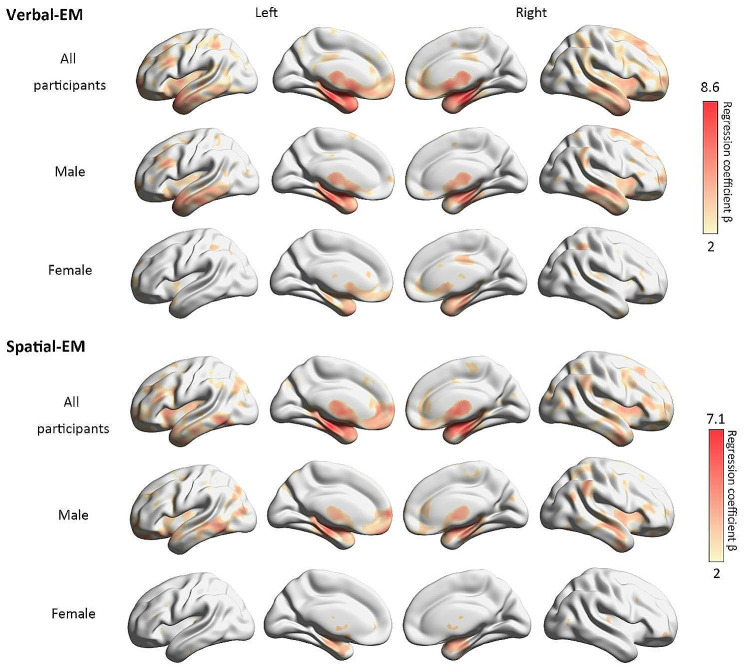
The brain regions significantly associated with two types of EM Only the areas with significant positive results are shown. The color bar shows the range of the regression coefficient β, with red indicating the strongest correlation and yellow indicating the weakest effect.

**Fig. 2:**
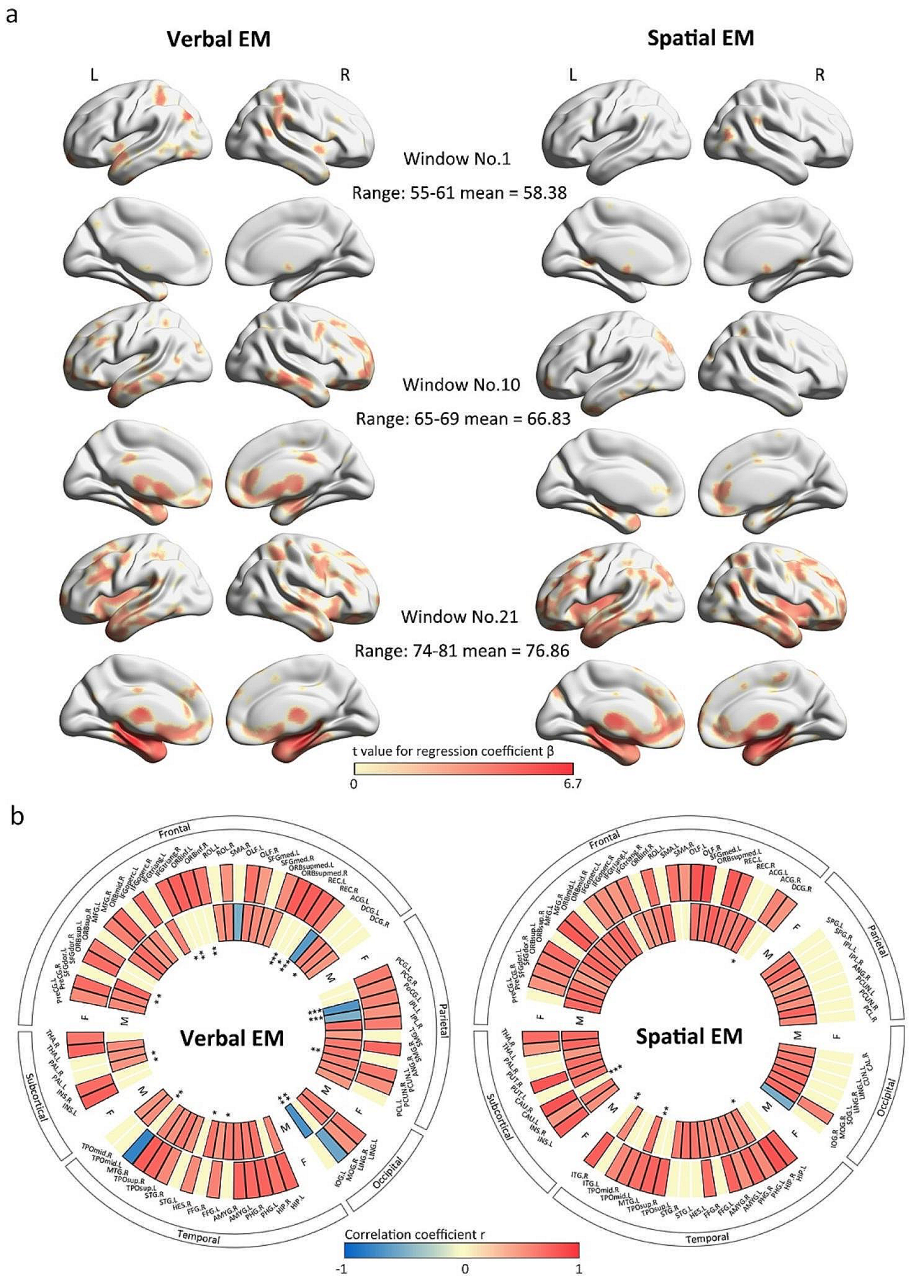
Age differences in the correlation between GMV and two types of EM. (a) Age differences in the t values indexing the relationship between GMV and two types of EM for the total sample. (b) A heatmap of the correlations between age and the strength of the GMV- EM association across five clusters of 90 brain regions. Males (outer circle) and females (inner circle) were analyzed separately. Each color block represents a brain region, with yellow blocks indicating nonsignificant regions, red blocks indicating regions with positive correlations, and blue blocks indicating regions with negative correlations. The names of the brain regions are displayed in the legend on the outermost circle. The innermost asterisks indicate the significance of differences in correlation coefficients (* < 0.05, ** < 0.01, *** < 0.001). Only regions where GMV-EM correlations with age were significant in either the male or female group, or in both groups, are displayed. In regions where there are no significant results for GMV-EM, the significance of differences cannot be calculated (for example, in the female group in SEM, the parietal and occipital lobes).

**Fig. 3:**
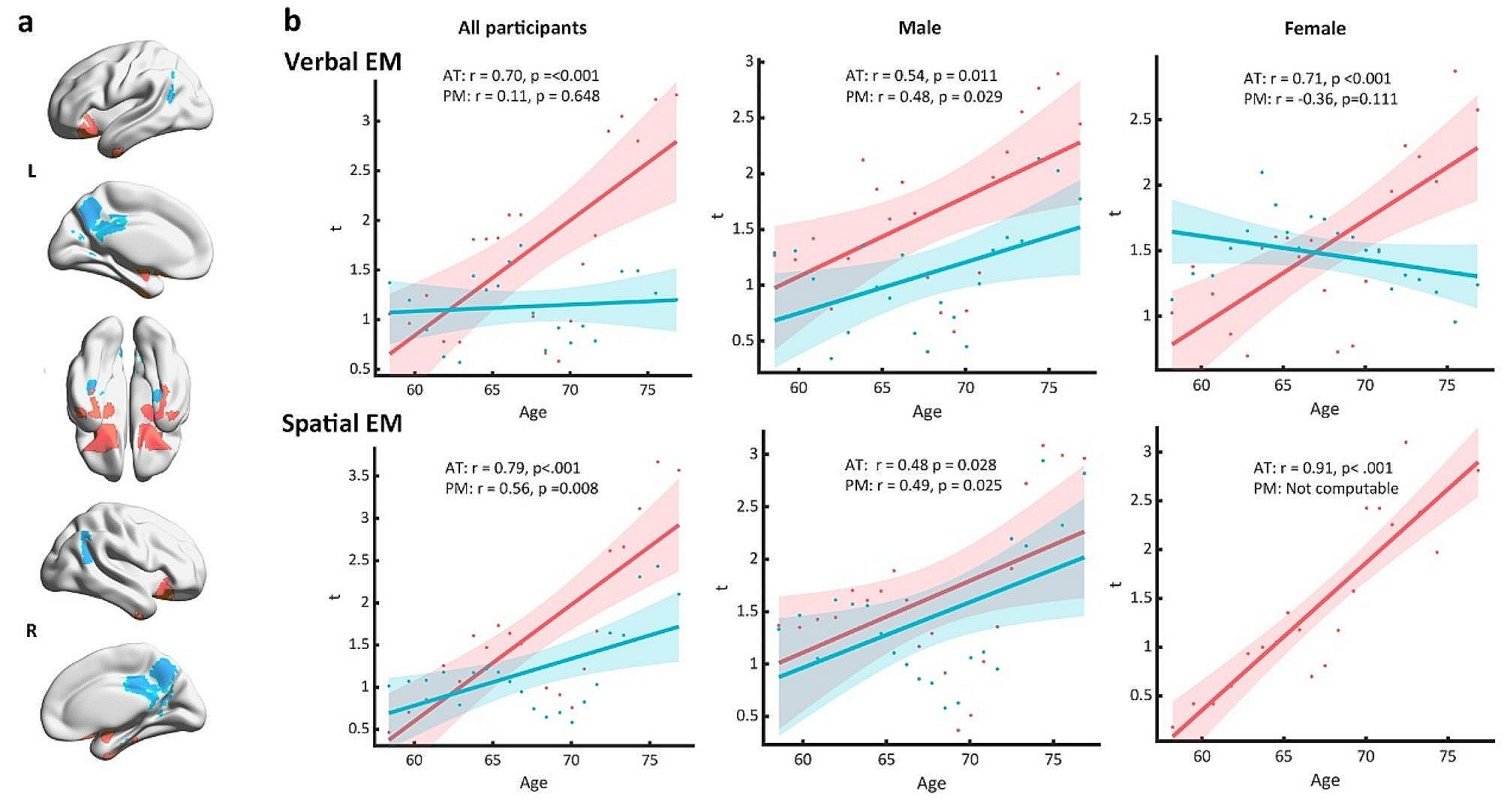
Age differences in VEM’s and SEM’s association with GMV of the two brain systems (a) Graphical representation of the AT and PM systems, with red representing the AT system and blue representing the PM system. (b) The age-related differences in VEM and SEM in relation to GMV in two brain systems, with each point in the figure representing the average t value within either the AT system (red) or the PM system (blue) for each window.

The analysis of the total sample found significant correlations of GMV and VEM in a wide range of cortical and subcortical regions, primarily located in the bilateral hippocampus, para-hippocampus, bilateral anterior cingulate gyrus, bilateral insula, bilateral thalamus, and bilateral amygdala. The parahippocampal gyrus showed the strongest correlation (Fig. 1, 1st row; Table S1 in the supplementary material). When analyzed separately for males and females, the results for males were similar to those of the total sample, except that the left amygdala showed the strongest effect (Figs. 1 and 2nd row; Table S2 in the supplementary material). For females, however, there were fewer significant regions, and the strongest effect was found in the temporal pole and middle temporal gyrus (Figs. 1 and 3rd row; Table S3 in the supplementary material).

#### SEM

**Fig. 4:**
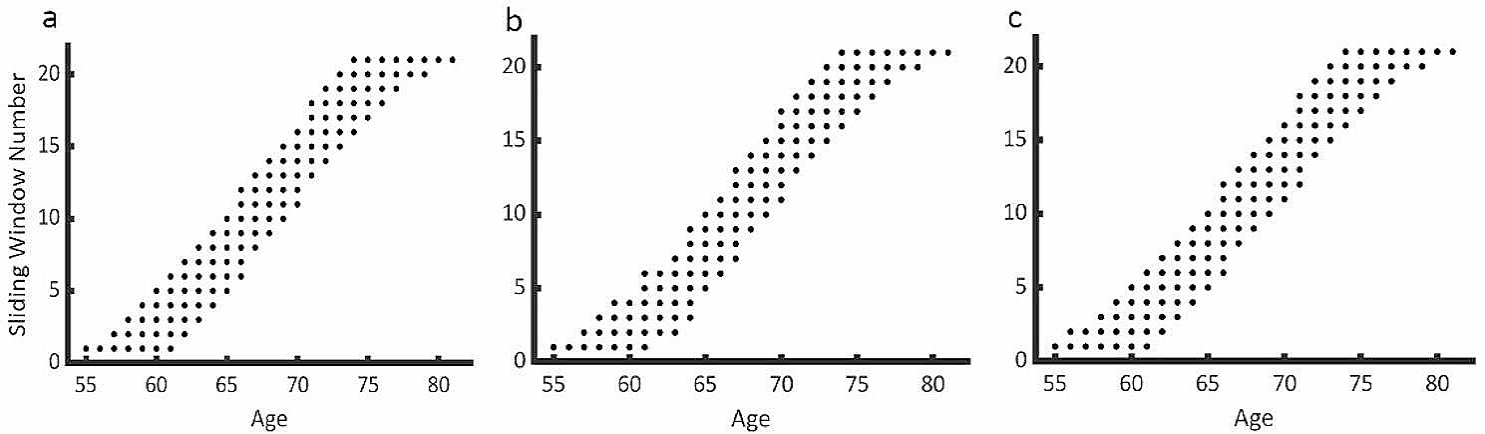
Age distribution in sliding windows for the total sample (a), males (b), and females (c)

For the total sample, we found significant correlations of GMV and SEM in a wide range of cortical and subcortical areas dominated by the medial temporal and frontal lobes, such as the bilateral hippocampus, para-hippocampus, bilateral anterior cingulate gyrus, bilateral insula, bilateral thalamus, bilateral amygdala, and a few parietal and occipital regions. The strongest correlation was found in the left hippocampus (Figs. 1 and 4th row, Table S4 in the supplementary material). The analysis of the males found relevant regions similar to those found for the total sample, with the strongest effect in the left hippocampus (Figs. 1 and 5th row, Table S5 in the supplementary material). Females again showed fewer and weaker effects than the males, and the strongest effect was observed in the temporal pole and superior temporal gyrus (Figs. 1 and 6th row, Table S6 in the supplementary material).

### Age differences in the relationship between GMV and two types of EM

The sliding windows approach was used to investigate age differences in the relationship between GMV and EM. Our first step was to generate binarized masks of the brain regions that showed significant relationship with each type of EM (refer to results 2.3 for details). The next step involves conducting multiple linear regressions for the group normalized residuals (referring to the residuals of GMV after regressing out covariates, standardized as z-scores across all samples) of GMV (controlling for age, education years, TIV, excluding sex only for all subject group) using two different EM within all windows, respectively. Subsequently, the average t value of the t-test statistic for the regression coefficients β (*p* < 0.01, uncorrected) within each region from AAL90 served as a measure of the association between EM and GMV within the AAL90 region [39]. The mean t-value within a region of each window was then correlated (Pearson’s correlation) with average age of each window, FDR = 0.05 corrected. Fisher’s z-Tests were then utilized to assess the significance of differences in the correlation coefficients (r) between the two sexes.

#### VEM

For the total sample, VEM was positively associated with GMV in large areas of the frontal and temporal lobes, as well as in smaller areas of the parietal, occipital, and subcortical lobes (see Fig. 2a). but those involving left superior parietal gyrus, right supramarginal gyrus, left angular, left inferior and middle occipital gyrus, and right putamen were negatively correlated with age (also see Table S7 in the supplementary material).

When analyzed by sex, somewhat different results were found by type of EM and sex. Results are summarized in heatmaps (see Fig. 2b), a different manner of presentation than that for the total sample. For males, the strength of the GMV-VEM association positively correlated with age in regions dominated by frontal, temporal, and parietal lobes, with some occipital and subcortical regions, but were negatively correlated with age in certain regions such as right rectus, right supplementary motor area, left postcentral, left inferior parietal gyrus, and left inferior occipital gyrus (Fig. 2b left outermost circle and Table S7 in the supplementary material). For females, positive correlations between the GMV-VEM association and age were found in extensive areas dominated by frontal, temporal, and parietal lobes, as well as some occipital and subcortical areas, but negative correlations were found in the left middle occipital gyrus and right middle temporal gyrus (Fig. 2b left innermost circle and Table S7 in the supplementary material). The results of the comparison of correlation coefficients in the two sex groups are presented in the left portion of Fig. 2b and Table S7 in the supplementary material.

#### SEM

For the total sample, SEM was positively associated with GMV in extensive areas dominated by frontal, temporal, parietal, and subcortical regions, as well as a small number of occipital regions, and the strength of these associations positively correlated with age (Table S8 in the supplementary material).

When analyzed by sex, we found that in males, the strength of the GMV-SEM association positively correlated with age in large areas of temporal and frontal lobes, as well as some parietal, occipital, and subcortical lobes, but negatively correlated with age in the right inferior occipital gyrus (Fig. 2b right outermost circle and Table S8 in the supplementary material). For females, we found strong positive correlations between age and the strength of the GMV-SEM association in various regions, mainly in the temporal lobe, and some weak positive correlations in frontal, occipital, and subcortical lobe. No region showed a negative correlation in females. The results of the comparison of correlation coefficients in the two sex groups are presented in the right portion of Fig. 2b and Table S7 in the supplementary material.

### Analysis at the level of brain systems: the AT and PM systems

Finally, we analyzed the data at the level of brain systems—the AT and PM systems, as discussed in the Introduction. In light of the t values calculated for each window as outlined in Sect. 2.4, the average t values were extracted for each type of EM within the AT and PM systems. Subsequently, Pearson correlation analyses were conducted to assess the relationship between average age of each window and average t values within each system of each window, both within the entire sample and separately for males and females. Fisher’s z-Tests were then utilized to assess the significance of differences in the correlation coefficients (r) between the two sexes.

#### VEM

For the total sample, age was positively correlated with the strength of the association between VEM and the GMV of the AT system (*r* = 0.70, *p* < 0.001), but not that between VEM and the GMV of the PM system (*r* = 0.11, *p* = 0.65) (Fig. 3b upper, left). In the analysis based on sex, distinct patterns emerged. Among males, the strength of VEM’s association with GMV in the AT system (*r* = 0.54, *p* = 0.011) and the PM system (*r* = 0.48, *p* = 0.029) both showed significant positive correlations with age. (Fig. 3b upper, middle). In females, however, age was significantly and positively associated with the strength of VEM’s association with GMV of the AT system (*r* = 0.71, *p* < 0.001), but not significantly associated with GMV of the PM system (*r* = -0.36, *p* = 0.111) (Fig. 3b upper, right). The sex differences in the correlation coefficient (r) for both systems are highly significant, as evidenced by the following statistical results: AT (Fisher’s Z = -4.29, *p* < 0.001) and PM (Fisher’s Z = 13.65, *p* < 0.001).

#### SEM

For the total sample, age was positively associated with the strength of SEM’s association with GMV of both the AT (*r* = 0.79, *p* < 0.001) and PM (*r* = 0.56, *p* = 0.008) systems (Fig. 3b below, left). When analyzed by sex, different patterns were observed. Similar to VEM, in males, the strength of the association between VEM and GMV in both the AT system (*r* = 0.48, *p* = 0.028) and the PM system (*r* = 0.49, *p* = 0.025) showed significant positive correlations with age (Fig. 3b below, middle). Females showed a strong positive correlation between age and the strength of SEM’s association with GMV of the AT system (*r* = 0.91, *p* < 0.001). There was no relevant finding for the PM system because no region within the PM system was significantly associated with females’ SEM scores (Fig. 3b below, right). The sex difference in the correlation coefficient (r) of the AT system is significant (Fisher’s Z = -9.71, *p* < 0.001). In contrast, the PM system cannot be compared due to the lack of statistical significance in the average t values for females across all windows.

## Discussion

Our study examined age-related differences in EM and GMV and their association in a Chinese sample of older community residents. The results indicated a negative correlation between EM and age, and that relevant brain regions included areas in the medial temporal lobe, frontal lobe, other neocortex, and subcortical regions. More importantly, we found that the relationship between EM and GMV depended on age, type of EM, and brain area/system. For the total sample, positive correlations were found between both types of EM and GMV in a number of brain regions located in the temporal and frontal lobes, and these associations positively correlated with age. When analyzed by sex, the association between VEM and GMVs in the insula and parietal regions became stronger with age for females but not males, whereas the association between SEM and GMVs in the parietal and occipital regions became stronger in males but not in females. When analyzed at the brain system level, we found that for males, there were positive age-related correlations in the associations between EM (either VEM or SEM) and the GMV of either the AT system or the PM system. For females, however, both VEM’s and SEM’s association with GMV became stronger with age in the AT system, but VEM’s association with GMV did not show a significant correlation with age in the PM system. The sex-specific patterns of age-related correlations in the association between two types of EM and specific regional GMV help elucidate the structural basis of EM for males and females in older individuals.

EM exhibit a negative correlation with age in older individuals is consistent with previous studies [40–42]. We further found that although females showed significantly lower SEM but higher VEM than did males. However, there was no significant difference between sexes in the correlation coefficient of EM scores and age for the two types of episodic memory, thus confirming previous studies [43]. These results suggest that sex differences in performance on different types of EM tasks are due to inherent differences between sexes, not the age [25, 26].

Our findings of the neural basis of EM are consistent with previous studies. Relevant brain areas included the medial temporal lobe and frontal lobe, as well as some regions of the parietal and occipital lobes, and subcortical areas [5, 7, 9, 11–14]. In terms of the two types of EM, VEM involves language pathways and auditory processing in the temporal and frontal lobes [44], whereas SEM involves visual-spatial processing in the parietal-frontal and parietal-medial temporal pathways [45]. We also found sex differences in the neural basis of EM. Specifically, compared to females, males show greater activity in brain regions associated with both VEM and SEM, consistent with a study by Sang, Chen [46].

In terms of the association between EM and GMV, our study yielded several important findings. First, for the total sample, the EM-GMV association typically becomes stronger with age. Specifically, the association between GMV and VEM positively correlated with age significantly in a wide range of regions in the frontal and temporal lobes, and a small number of regions in the parietal lobe, occipital lobe, and subcortical lobes. These regions are responsible for normal cognitive performance and have been linked to VEM in previous studies [4–8, 11]. Similarly, the association between GMV and SEM positively correlated with age significantly in a similar regions as those for VEM, but involved more areas in the parietal lobe, occipital lobe, and subcortical regions than did VEM, perhaps because SEM involves more visual and spatial information than does VEM [45]. Furthermore, our findings demonstrate a greater number of brain regions positively correlated with SEM comparison to VEM, specifically within the parietal and occipital lobes. These areas are associated with the processing of visual spatial information. Gorbach, Pudas [6] speculated that after the age of 65, both brain biomarkers and cognitive measurements show more pronounced changes, leading to stronger associations between these changes. Furthermore, some studies have also found that age can induce variability in gray matter structure [47, 48]. In our supplementary analysis, we computed the inter-subject variability of GMV within each window. There is a growing trend in variability with age, although it did not reach statistical significance (Fig. S11). Our findings provide further supplementation to this perspective, indicating a positive correlation between age and association between GMV and EM, which manifests with specificity in various types of EM.

Second, there are notable differences between males and females in the association between EM and GMV in older individuals. Specifically, for VEM, males show significantly lower age-related correlation between GMV-VEM in many regions in the frontal and parietal lobes, as well as subcortical areas (e.g., right superior frontal gyrus, bilateral inferior frontal gyrus, triangular part, left precuneus, bilateral insula, etc.). However, in the temporal lobe, males exhibit more regions with significantly higher correlations with age (e.g., right amygdala, right fusiform gyrus, right middle temporal gyrus, and left middle temporal gyrus). In the regions where females show significant differences in correlations, many of them are associated with language, such as bilateral inferior frontal gyrus, triangular part, left superior parietal gyrus and left inferior parietal gyrus [49, 50]. For SEM, males exhibit significant positive correlations between GMV-VEM and age in most regions of the parietal and occipital lobes (e.g., bilateral superior parietal gyrus, left superior parietal gyrus, left angular gyrus, right precuneus, right lateral occipital cortex, right calcarine, left cuneus, bilateral lingual gyrus, and left superior occipital gyrus). In these areas, there is a strong association with visual and spatial information processing [51–53]. On the other hand, females do not show significant GMV-VEM correlations in these regions, making it impossible to compare correlation coefficients. Instead, females demonstrate more regions with significantly lower correlations (e.g., right hippocampus, right superior temporal gyrus, right middle temporal gyrus). To further probe sex differences, we also examined age-related variations in GMV in the two sex groups (see Supplementary Material Table S9). Overall, for both sexes, the GMV in the majority of brain regions were negatively correlated with age, except for the right putamen and bilateral pallidum. Direct comparisons between the two sexes showed that, in most regions (14 regions), a greater extent of negative correlation with age compared to females (as indicated by larger absolute values of males’ t values in the regression analysis). This sex difference in age-related variations in GMV may account for the greater GMV-EM correlations for males as compared to females, which in turn may explain sex differences in VEM and SEM. The differences in these correlation patterns may stem from varying differences in GMV to support different cognitive advantages in older individuals of different sexes, resulting in distinct GMV-EM association patterns. These can be validated in longitudinal studies. The potential causes of sex differences may stem from various factors, including evolutionary demands, hormonal and endocrine influences, and the heterogeneity of older individuals. Firstly, from an evolutionary standpoint, these differences likely have an evolutionary basis related to sexual selection pressures [27, 54]. Secondly, sex hormones modulate brain structure and function through different receptors or pathways, affecting mechanisms such as neurogenesis, dendritic spine density, and synaptic plasticity [27]. Thirdly, brain structural variability between individuals tends to increase with age [47, 48]. In our supplementary analysis, we conducted correlation analyses between the variability of GMV and age among participants of different sexes. The results indicated a significant positively correlation in variability with age among males, whereas females exhibited the opposite trend. This suggests that heterogeneous differences may not be the sole age-related factor influencing the relationship between GMV and EM. We further explored the impact of different overlaps on the GMV-EM relationship with age based on sliding windows. Specific results can be found in Fig S12 (70% overlap) and Fig. 13 (60% overlap). We observed that smaller overlaps resulted in a reduced number of delineated windows along the age axis. Despite the challenge posed by the decreased window count, the positive correlation between GMV-EM and age remained stable.

Finally, we investigated sex differences in age-related variations in the association between EM and GMV at the level of two brain systems (AT and PM). At the entire sample, significant positive correlations with age were observed for both VEM-GMV and SEM-GMV associations with the AT and PM systems. However, VEM-GMV association with the PM system did not exhibit a significant correlation with age. Sex-specific analyses showed that in males, both types of EM demonstrated a significant positive correlation with age in both systems. In females, both types of EM displayed a significant positive correlation with age in the AT system, while VEM’s association with the PM system did not significantly correlate with age. Notably, the SEM-GMV association with the PM system did not yield significant results across age groups. Differences in correlation coefficients revealed that females had significantly higher correlations with age for both types of EM in the AT system compared to males. Conversely, in the PM system, the GMV-VEM relationship showed significantly lower correlations with age in females compared to males. These findings suggest sex differences in the age-related patterns of the relationships between EM types and GMV in the AT and PM systems, with males receiving stronger support from both systems, and females showing a stronger inclination toward the GMV of the AT system. The AT system, associated with processing verbal information, and the PM system, inclined towards handling visual-spatial information [16]. Our analysis focused exclusively on regions positively correlated with EM. Building upon the observed sex-specific patterns mentioned earlier, it may unveil that the consistent predominance of stronger SEM in males is linked to a more robust correlation of the GMV of the PM system with age. Conversely, the stable manifestation of stronger VEM in females is associated with a stronger correlation of the GMV of the AT system with age. We offer a new perspective on previous functional studies of the AT and PM systems and EM from the standpoint of GMV [55, 56]. In summary, our findings suggest a higher correlation between GMV-EM in regions associated with the EM in the elderly population, indicating that GMV is more supportive of cognitive performance in older individuals.

In summary, we observed stronger correlation between EM and specific region GMV in the elderly population. Previous research indicates that one of the hallmarks of brain aging is dedifferentiation [57], suggesting a convergence of function across a wide range of brain areas. Our findings reveal that with older age GMV exhibits a broader and stronger correlation with EM, consistent with the phenomenon of brain functional dedifferentiation. Further exploration of this phenomenon necessitates utilizing multimodal data in subsequent studies to investigate its underlying mechanisms.

Several limitations of this study need to be mentioned. First, our age-related conclusions were based on cross-sectional data. They should be validated with large-scale longitudinal data in future studies. Second, the male sample in our data had more years of education than did the female sample. We used statistical control to handle this issue, but we did not explore how sampling methods and education policies affected the characteristics of our sample and the results. Thirdly, our study did not encompass additional variables such as lifestyle habits, diseases, biological information, etc., which can influence the causes of cognitive aging. These valuable variables will be taken into account in future research to provide a more comprehensive characterization of the relationship between cognition and brain structure.

## Conclusions

Our study found that the associations between EM and GMV in several brain regions positively correlated significantly with age, suggesting significant differences in the structural basis of cognitive functions in older adults. Furthermore, we observed significant sex disparities in these age-related variations, which were validated within the framework of the AT and PM systems. This suggests sex differences in addressing age-related variations. These findings should lead to a better understanding of the sex and age differences in neural bases of EM impairment in older individuals, which in turn should facilitate the discovery of more effective biomarkers and earlier diagnosis of dementia, and ultimately improve the health of older adults.

## Method

### Participants

The participants in this study were drawn from the cross-sectional data of the BABRI [58], an ongoing cohort study of brain and cognitive in community-dwelling older adults in Beijing, China. The inclusion and exclusion criteria were that all participants (1) were native Chinese speakers aged over 50 years old, without dementia and with normal daily living skills; (2) had no brain tumors, neurological or psychiatric disorders, or history of addiction; (3) had no known diseases that affect brain function, including alcoholism, current depression, Parkinson’s disease, or epilepsy; and (4) had no contraindications to magnetic resonance imaging (MRI). Based on this, we further excluded subjects with MMSE scores less than 24 and a clinical diagnosis of dementia. After applying these criteria and excluding data with missing demographic information, a remaining dataset was randomly sampled, consisting of 463 females and 463 males aged between 55 and 90 years. Basic information about the participants is presented in Table 1. The age distribution of the sample used in the study is presented in Fig S10 in the supplementary material.

### EM tests

EM was assessed by the auditory verbal learning test (AVLT) [59] and Rey-Osterrieth complex figure test (ROCF) [60]. In this study, we used the standardized scores (Z scores) of the total score of AVLT (AVLT-total) and ROCF-delay to index the performance of VEM and SEM, respectively.

### Image acquisition

T1-weighted image of each subject was acquired. Participants laid in a supine position with their heads fixed snugly by straps and foam pads to minimize head movement. T1-weighted, sagittal 3D magnetization-prepared rapid gradient echo (MP-RAGE) sequences were acquired with the following parameters (covering the entire brain): 176 sagittal slices, repetition time (TR) = 1900 ms, echo time (TE) = 3.44 ms, slice thickness = 1 mm, flip angle = 9°, inversion time = 900 ms, field of view (FOV) = 256 × 256 mm^2^, and acquisition matrix = 256 × 256.

### Imaging processing

Voxel-Based Morphometry (VBM) volume estimation for T1-weighted images was conducted using the Computational Anatomy Toolbox (CAT12) within the MATLAB (2020b) platform (http://dbm.neuro.uni-jena.de/cat12/). First, we performed voxel-based initial processing on the image, including denoising filtering, resampling, bias correction, image standardization, and initial segmentation using TPM. Second, the images of initial voxel-based processing were used for tissue segmentation of the gray matter, white matter, and cerebrospinal fluid, then local intensity correction, and segmentation were performed using Adaptive Maximum A Posterior (AMAP). Third, images were registered to the standard space of the Montreal Neurological Institute (MNI) by using modulated normalization. Finally, Gaussian smoothing was performed using 8 mm half-maximized full width (FWHM) smoothing for normalized gray matter image files.

### Statistical analyses

To investigate the relationship between GMV and EM and how it varies with age, we performed the following statistical analyses. First, regression imputation was used to replace the missing values of the EM tests. The percentage of missing data for VEM was 0.3% (3 cases) and for SEM, it was 1.4% (13 cases), indicating a relatively low proportion of missing values. Regression imputation was performed using SPSS 22.0 software, with separate imputations conducted for the male and female groups. The predictor variables used for imputation were age and education. Second, we converted the original scores of the EM tests to the Z scores for subsequent analyses. Third, we ran multiple linear regression analyses to examine age-related variations in VEM and SEM after controlling for years of education (as well as sex in the analysis of the total sample) by using R language [61]. Fourth, multiple linear regression was employed to investigate the relationship between GMV, and standardized VEM or SEM scores, separately. The models included age, years of education, and total intracranial volume (TIV) as covariates (Formula 1). Additionally, in the analysis involving the entire sample, sex was included as an additional covariate (Formula 2). In this study, our focus is solely on the positive correlation between GMV and EM. Consequently, we only present results where β is greater than 0. The analyses were conducted using MATLAB (2020b) and SPM12 (http://www.fil.ion.ucl.ac.uk/spm/software/spm12/).

$$\begin{aligned}{\rm Formula \;1}{:} E{M_{Norm}} & = {\beta _0}\, + {\beta _1}\,GMV\, + \,{\beta _2}age\, + \\ & {\beta _3}education\,years\, + \,{\beta _4}\,TIV\, + \,e \end{aligned}$$

$$\begin{aligned}{\rm Formula \;2}{:} \,E{M_{Norm}}\, &= {\beta _0}\, + \,{\beta _1}GMV\, + {\beta _2}age\, \\ &+ \,{\beta _3}\,education\,years\, + {\beta _4}\,TIV\\ &+ \,{\beta _5}\,sex\, + \,e \end{aligned}$$

Finally, to describe in a continuous manner the age differences in the strength of GMVs’ associations with VEM and SEM, we carried out the sliding window analysis (for the total sample: width = 180, overlap = 80%; for males and females separately: width = 90, overlap = 80%). Specifically, within each window, before applying formula 5, we standardized the normalized GMV residuals ${\widehat{e}}_{Norm}$​ through z-score standardization of $\widehat{e}$ in formula 3 and 4 (controlling for age, education years, TIV for both sex groups with Formula 3, and excluding sex only for the entire sample with Formula 4). Subsequently, we conducted simple linear regressions using Formula 5 on these normalized GMV residuals. These regressions utilized two different normalized EM scores as separate dependent variables. The same tools as in the previous step were utilized.

$$\begin{aligned} {\rm Formula\, 3{:}} \;GMV\, & = {\gamma _0}\, + \,{\gamma _1}\,age\, \\ &+ {\gamma _2}\,education\,years\, + \,{\gamma _3}\,TIV + e \end{aligned}$$

$$\begin{aligned} {\rm Formula\, 4{:}} \;GMV&= {\gamma }_{0} + {\gamma }_{1}age \\&+ {\gamma }_{2}education years +{\gamma }_{3}TIV \\&+ {\gamma }_{4}sex + e\end{aligned}$$

$${\rm Formula\, 5{:}} \; E{M}_{Norm}={\delta }_{0}+ {\delta }_{1}{\widehat{e}}_{Norm}+u$$

In the analysis mentioned above, the correction for FDR was applied using MATLAB’s mafdr function. We then employed t value of t-test statistic for the regression coefficients ${\delta }_{1}$for subsequent analysis. The AAL90 template [39], the AT and PM systems [16] were employed to extract the average t-values within each ROI, followed by a Pearson correlation analysis with the respective average age of each window to describe their age-related variations. Fisher’s z-Tests were then utilized to assess the significance of differences in the correlation coefficients (r) between the two sexes. A visual representation of the data for a sliding window configuration is given in Fig. 4.

### Electronic supplementary material

Below is the link to the electronic supplementary material.

Supplementary Material 1

## Data Availability

The datasets generated and/or analyzed during the current study are not publicly available due to the temporary non-disclosure of this dataset, but they can be obtained from the corresponding author upon reasonable request.
