# Supplementary material for: Age-related enhancement of the association between episodic memory and gray matter volume in medial temporal and frontal lobes
Source: Behav Brain Funct. 2024 May 3;20:10. doi: 10.1186/s12993-024-00237-y (PMC11069137; doi:10.1186/s12993-024-00237-y)
Supplement: Supplementary file 1 — Supplementary Material 1 [file 12993_2024_237_MOESM1_ESM.docx]

| **Table S1 Brain regions associated with VEM score in all subjects** | | | | | |
| --- | --- | --- | --- | --- | --- |
|  |  | Peak Voxel Coordinate | | |  |
| AAL Region of Peak Voxel | Cluster size | x | y | z | T |
| ParaHippocampal_R | 78556 | 28.5 | -19.5 | -19.5 | 8.5189 |
| Frontal_Inf_Orb_L | 165 | -39 | 37.5 | -6 | 4.1553 |
| Temporal_Sup_R | 28 | 60 | -1.5 | 3 | 3.343 |
| Insula_R | 69 | 36 | -19.5 | 10.5 | 3.5599 |
| Temporal_Mid_L | 135 | -57 | -58.5 | 16.5 | 3.7546 |
| Frontal_Sup_R | 2409 | 25.5 | 27 | 52.5 | 5.2929 |
| Temporal_Sup_R | 102 | 61.5 | -55.5 | 21 | 3.4946 |
| Cingulum_Mid_L | 412 | -4.5 | 24 | 34.5 | 3.725 |
| Temporal_Sup_L | 310 | -58.5 | -36 | 21 | 3.784 |
| Frontal_Inf_Tri_L | 2020 | -42 | 24 | 28.5 | 5.5511 |
| Frontal_Sup_L | 581 | -18 | 52.5 | 25.5 | 4.1453 |
| Occipital_Sup_R | 26 | 28.5 | -84 | 27 | 3.2975 |
| Frontal_Inf_Oper_R | 61 | 40.5 | 19.5 | 30 | 3.5983 |
| Cingulum_Mid_R | 984 | 3 | -19.5 | 43.5 | 4.6377 |
| SupraMarginal_R | 31 | 66 | -46.5 | 33 | 3.4554 |
| Precentral_R | 145 | 60 | 1.5 | 40.5 | 3.8365 |
| SupraMarginal_R | 64 | 57 | -43.5 | 43.5 | 3.5598 |
| Parietal_Inf_L | 570 | -34.5 | -42 | 49.5 | 4.9553 |
| Cingulum_Mid_L | 111 | -1.5 | -4.5 | 48 | 3.6414 |
| Postcentral_L | 273 | -52.5 | -10.5 | 54 | 4.1035 |
| Precentral_L | 23 | -31.5 | -7.5 | 58.5 | 3.2918 |
| Frontal_Sup_R | 355 | 25.5 | -7.5 | 66 | 4.2598 |
| Frontal_Sup_L | 229 | -25.5 | 9 | 66 | 4.4347 |
| Paracentral_Lobule_L | 68 | -3 | -15 | 73.5 | 3.462 |

FDR<.05 corrected, Cluster size≥10; T represents the statistical value of the t-test for the regression coefficient β corresponding to the peak voxel.

| **Table S2 Brain regions associated with VEM score in the male group** | | | | | |
| --- | --- | --- | --- | --- | --- |
|  |  | Peak Voxel Coordinate | | |  |
| AAL Region of Peak Voxel | Cluster size | x | y | z | T |
| Amygdala_L | 53810 | -28.5 | -1.5 | -18 | 7.4418 |
| Frontal_Sup_Orb_R | 831 | 18 | 40.5 | -15 | 4.2503 |
| Fusiform_R | 44 | 43.5 | -66 | -19.5 | 3.5546 |
| Frontal_Sup_Medial_R | 42 | 3 | 45 | 0 | 3.5541 |
| Insula_L | 160 | -39 | 6 | 1.5 | 3.8684 |
| Frontal_Mid_R | 711 | 27 | 58.5 | 3 | 5.0584 |
| Frontal_Inf_Tri_L | 27 | -43.5 | 42 | 3 | 3.2202 |
| Frontal_Inf_Tri_R | 119 | 46.5 | 30 | 9 | 3.523 |
| SupraMarginal_R | 1854 | 55.5 | -43.5 | 43.5 | 4.7515 |
| Temporal_Sup_R | 22 | 61.5 | -18 | 10.5 | 3.4064 |
| Temporal_Mid_L | 206 | -58.5 | -57 | 16.5 | 4.1886 |
| Frontal_Inf_Tri_L | 902 | -43.5 | 22.5 | 28.5 | 5.516 |
| Frontal_Inf_Tri_R | 94 | 39 | 19.5 | 28.5 | 3.6837 |
| Frontal_Inf_Oper_R | 34 | 49.5 | 12 | 30 | 3.4882 |
| Frontal_Mid_L | 1102 | -36 | 31.5 | 48 | 5.0189 |
| Frontal_Sup_R | 128 | 16.5 | 55.5 | 34.5 | 3.9495 |
| Occipital_Mid_R | 23 | 36 | -67.5 | 34.5 | 3.4752 |
| Frontal_Sup_R | 1434 | 25.5 | 27 | 52.5 | 5.7343 |
| Cingulum_Mid_L | 112 | -3 | -18 | 39 | 3.793 |
| Parietal_Inf_L | 20 | -33 | -40.5 | 49.5 | 3.4962 |
| Supp_Motor_Area_R | 98 | 7.5 | -1.5 | 51 | 3.7467 |
| Parietal_Sup_L | 117 | -34.5 | -48 | 66 | 3.6621 |
| Frontal_Mid_L | 195 | -25.5 | 7.5 | 64.5 | 4.1708 |
| Parietal_Sup_R | 123 | 36 | -54 | 60 | 3.7503 |
| Supp_Motor_Area_R | 1221 | 16.5 | -3 | 69 | 4.6321 |
| Parietal_Sup_R | 12 | 13.5 | -70.5 | 64.5 | 3.3753 |
| Parietal_Sup_L | 17 | -16.5 | -64.5 | 67.5 | 3.4757 |
| Paracentral_Lobule_L | 29 | -3 | -16.5 | 73.5 | 3.3461 |

FDR<.05 corrected, Cluster size≥10; T represents the statistical value of the t-test for the regression coefficient β corresponding to the peak voxel.

|  | | | | | |
| --- | --- | --- | --- | --- | --- |
| **Table S3 Brain regions associated with VEM score in the female group** | | | | | |
|  |  | Peak Voxel Coordinate | | |  |
| AAL Region of Peak Voxel | Cluster size | x | y | z | T |
| Temporal_Pole_Mid_L | 10227 | -28.5 | 9 | -42 | 5.2001 |
| Hippocampus_R | 9789 | 28.5 | -18 | -18 | 5.4467 |
| Frontal_Sup_Orb_R | 70 | 15 | 43.5 | -19.5 | 3.4186 |
| Lingual_R | 211 | 9 | -69 | -12 | 4.2354 |
| Frontal_Mid_Orb_L | 206 | -30 | 54 | -12 | 3.7138 |
| Frontal_Inf_Orb_R | 226 | 48 | 28.5 | -4.5 | 3.5644 |
| Precuneus_L | 39 | -12 | -39 | 6 | 3.4678 |
| Frontal_Sup_L | 210 | -19.5 | 67.5 | 6 | 3.6999 |
| Thalamus_R | 173 | 7.5 | -13.5 | 10.5 | 3.5358 |
| Heschl_R | 65 | 39 | -22.5 | 10.5 | 3.3295 |
| Frontal_Inf_Oper_R | 10 | 55.5 | 13.5 | 12 | 3.2274 |
| Frontal_Mid_R | 134 | 52.5 | 42 | 13.5 | 4.1931 |
| Thalamus_L | 14 | -6 | -13.5 | 12 | 3.1685 |
| Temporal_Mid_R | 16 | 46.5 | -49.5 | 12 | 3.1766 |
| Occipital_Mid_R | 46 | 31.5 | -82.5 | 27 | 3.7127 |
| Cingulum_Mid_R | 741 | 7.5 | -16.5 | 36 | 4.3448 |
| Frontal_Sup_L | 21 | -18 | 52.5 | 27 | 3.2793 |
| Frontal_Sup_R | 38 | 18 | 43.5 | 30 | 4.0265 |
| Parietal_Inf_R | 991 | 37.5 | -43.5 | 49.5 | 5.0708 |
| Parietal_Inf_L | 551 | -37.5 | -42 | 49.5 | 4.107 |
| Frontal_Mid_R | 24 | 49.5 | 7.5 | 52.5 | 3.268 |
| Postcentral_L | 12 | -54 | -9 | 52.5 | 3.3617 |

FDR<.05 corrected, Cluster size≥10; T represents the statistical value of the t-test for the regression coefficient β corresponding to the peak voxel.

| \| **Table S4 Brain regions associated with SEM score in all subjects** \| \| \| \| \| \| \| --- \| --- \| --- \| --- \| --- \| --- \| \|  \|  \| Peak Voxel Coordinate \| \| \|  \| \| AAL Region of Peak Voxel \| Cluster size \| x \| y \| z \| T \| \| Hippocampus_L \| 46792 \| -27 \| -19.5 \| -16.5 \| 7.0684 \| \| Vermis_7 \| 44 \| 1.5 \| -76.5 \| -28.5 \| 3.4257 \| \| Frontal_Sup_Medial_L \| 6088 \| -6 \| 63 \| 1.5 \| 4.9166 \| \| Fusiform_L \| 97 \| -34.5 \| -78 \| -18 \| 3.6251 \| \| Frontal_Sup_Orb_R \| 140 \| 16.5 \| 55.5 \| -12 \| 3.6364 \| \| Frontal_Mid_Orb_R \| 126 \| 37.5 \| 51 \| -3 \| 3.4003 \| \| Temporal_Mid_R \| 841 \| 52.5 \| -63 \| 21 \| 4.3386 \| \| Frontal_Mid_R \| 89 \| 27 \| 58.5 \| 6 \| 3.4905 \| \| Temporal_Sup_R \| 139 \| 67.5 \| -21 \| 16.5 \| 3.6846 \| \| Frontal_Sup_Medial_R \| 43 \| 10.5 \| 63 \| 10.5 \| 3.5127 \| \| Frontal_Inf_Oper_L \| 10 \| -45 \| 7.5 \| 10.5 \| 3.2108 \| \| Occipital_Mid_R \| 295 \| 31.5 \| -87 \| 31.5 \| 4.2484 \| \| Temporal_Mid_L \| 22 \| -57 \| -60 \| 18 \| 3.3816 \| \| SupraMarginal_R \| 1007 \| 57 \| -45 \| 36 \| 3.9864 \| \| Precentral_L \| 374 \| -42 \| 4.5 \| 30 \| 4.654 \| \| Left Angular Gyrus \| 313 \| -46.5 \| -75 \| 34.5 \| 4.1248 \| \| Frontal_Inf_Oper_R \| 68 \| 42 \| 18 \| 33 \| 3.5112 \| \| Occipital_Sup_L \| 120 \| -24 \| -76.5 \| 42 \| 3.6085 \| \| Frontal_Sup_R \| 381 \| 25.5 \| 27 \| 51 \| 4.1512 \| \| Precentral_R \| 270 \| 48 \| 7.5 \| 45 \| 4.1983 \| \| Cingulum_Mid_R \| 20 \| 3 \| -7.5 \| 46.5 \| 3.2354 \| \| Parietal_Sup_R \| 172 \| 36 \| -52.5 \| 55.5 \| 4.2625 \| \| Postcentral_R \| 24 \| 58.5 \| -19.5 \| 52.5 \| 3.4109 \| \| Precentral_L \| 51 \| -30 \| -9 \| 58.5 \| 3.314 \| \| Supp_Motor_Area_R \| 13 \| 4.5 \| -28.5 \| 55.5 \| 3.1578 \| \| Parietal_Sup_R \| 43 \| 18 \| -66 \| 64.5 \| 3.4584 \| \| Frontal_Sup_L \| 35 \| -21 \| 7.5 \| 63 \| 3.2902 \| \| Frontal_Sup_R \| 11 \| 28.5 \| 4.5 \| 67.5 \| 3.5828 \| |
| --- | --- | --- | --- | --- | --- | --- | --- | --- | --- | --- | --- | --- | --- | --- | --- | --- | --- | --- | --- | --- | --- | --- | --- | --- | --- | --- | --- | --- | --- | --- | --- | --- | --- | --- | --- | --- | --- | --- | --- | --- | --- | --- | --- | --- | --- | --- | --- | --- | --- | --- | --- | --- | --- | --- | --- | --- | --- | --- | --- | --- | --- | --- | --- | --- | --- | --- | --- | --- | --- | --- | --- | --- | --- | --- | --- | --- | --- | --- | --- | --- | --- | --- | --- | --- | --- | --- | --- | --- | --- | --- | --- | --- | --- | --- | --- | --- | --- | --- | --- | --- | --- | --- | --- | --- | --- | --- | --- | --- | --- | --- | --- | --- | --- | --- | --- | --- | --- | --- | --- | --- | --- | --- | --- | --- | --- | --- | --- | --- | --- | --- | --- | --- | --- | --- | --- | --- | --- | --- | --- | --- | --- | --- | --- | --- | --- | --- | --- | --- | --- | --- | --- | --- | --- | --- | --- | --- | --- | --- | --- | --- | --- | --- | --- | --- | --- | --- | --- | --- | --- | --- | --- | --- | --- | --- | --- | --- | --- | --- | --- | --- | --- | --- | --- | --- | --- | --- |
| FDR<.05 corrected, Cluster size≥10; T represents the statistical value of the t-test for the regression coefficient β corresponding to the peak voxel. |

|  | | | | | |
| --- | --- | --- | --- | --- | --- |
| **Table S5 Brain regions associated with SEM score in the male group** | | | | | |
|  |  | Peak Voxel Coordinate | | |  |
| AAL Region of Peak Voxel | Cluster size | x | y | z | T |
| Hippocampus_L | 13725 | -27 | -19.5 | -16.5 | 5.7801 |
| Hippocampus_R | 15291 | 27 | -9 | -13.5 | 5.8842 |
| Temporal_Inf_L | 53 | -43.5 | 1.5 | -33 | 3.775 |
| Temporal_Inf_R | 80 | 52.5 | -48 | -25.5 | 3.4727 |
| Temporal_Inf_L | 4462 | -60 | -57 | -12 | 5.004 |
| Occipital_Inf_R | 160 | 40.5 | -67.5 | -13.5 | 3.8968 |
| Frontal_Sup_Orb_R | 10 | 13.5 | 66 | -15 | 3.2257 |
| Temporal_Sup_L | 566 | -55.5 | 3 | -1.5 | 4.1607 |
| Insula_L | 85 | -36 | 13.5 | 4.5 | 3.3609 |
| Temporal_Sup_R | 250 | 66 | -21 | 16.5 | 4.1511 |
| Insula_L | 283 | -40.5 | -10.5 | 10.5 | 3.7041 |
| Frontal_Sup_R | 26 | 28.5 | 58.5 | 7.5 | 3.4041 |
| Occipital_Mid_L | 850 | -37.5 | -85.5 | 13.5 | 4.4143 |
| Frontal_Sup_Medial_R | 19 | 9 | 63 | 10.5 | 3.3098 |
| Caudate_L | 32 | -9 | 6 | 15 | 3.2347 |
| Rolandic_Oper_L | 17 | -49.5 | 1.5 | 13.5 | 3.4233 |
| Angular_R | 642 | 54 | -64.5 | 24 | 4.5138 |
| Cuneus_R | 78 | 7.5 | -81 | 22.5 | 3.663 |
| Frontal_Inf_Tri_L | 32 | -49.5 | 27 | 22.5 | 3.4959 |
| SupraMarginal_R | 37 | 64.5 | -30 | 25.5 | 3.3303 |
| SupraMarginal_R | 1113 | 55.5 | -43.5 | 43.5 | 4.7001 |
| Precentral_L | 136 | -42 | 4.5 | 30 | 4.0936 |
| Frontal_Sup_L | 381 | -25.5 | 39 | 40.5 | 3.7175 |
| Occipital_Sup_L | 276 | -25.5 | -76.5 | 42 | 4.2384 |
| Frontal_Mid_R | 10 | 28.5 | 33 | 40.5 | 3.2824 |
| Frontal_Sup_Medial_L | 13 | -7.5 | 55.5 | 42 | 3.3225 |
| Precentral_R | 25 | 46.5 | 6 | 43.5 | 3.4095 |
| Frontal_Mid_R | 42 | 51 | 18 | 45 | 3.4995 |
| Parietal_Inf_L | 53 | -52.5 | -30 | 48 | 3.5699 |
| Parietal_Sup_R | 169 | 36 | -52.5 | 55.5 | 4.0348 |
| Precentral_L | 226 | -30 | -9 | 57 | 3.9478 |
| Postcentral_R | 28 | 34.5 | -36 | 57 | 3.2398 |
| Paracentral_Lobule_R | 15 | 4.5 | -33 | 55.5 | 3.2193 |
| Parietal_Sup_R | 107 | 18 | -67.5 | 66 | 4.134 |
| Precuneus_L | 44 | -7.5 | -63 | 70.5 | 3.3222 |
| Parietal_Sup_L | 58 | -21 | -39 | 63 | 3.5876 |
| Frontal_Sup_R | 16 | 28.5 | 4.5 | 67.5 | 3.5723 |
| Frontal_Sup_R | 29 | 16.5 | 0 | 73.5 | 3.4359 |

FDR<.05 corrected, Cluster size≥10; T represents the statistical value of the t-test for the regression coefficient β corresponding to the peak voxel.

|  | | | | | |
| --- | --- | --- | --- | --- | --- |
| **Table S6 Brain regions associated with SEM score in the female group** | | | | | |
|  |  | Peak Voxel Coordinate | | |  |
| AAL Region of Peak Voxel | Cluster size | x | y | z | T |
| Temporal_Pole_Sup_R | 4303 | 31.5 | 4.5 | -22.5 | 4.6369 |
| Temporal_Inf_L | 105 | -45 | -7.5 | -39 | 3.3824 |
| ParaHippocampal_L | 3651 | -30 | -18 | -24 | 4.4209 |
| Cerebelum_Crus2_L | 213 | -3 | -76.5 | -33 | 3.9143 |
| Temporal_Inf_R | 10 | 46.5 | -9 | -36 | 3.2274 |
| Temporal_Mid_L | 169 | -54 | -13.5 | -21 | 3.6852 |
| Fusiform_R | 447 | 42 | -36 | -15 | 4.4786 |
| Frontal_Sup_Orb_R | 30 | 12 | 46.5 | -25.5 | 3.403 |
| Olfactory_L | 343 | -9 | 9 | -15 | 3.9771 |
| Temporal_Inf_L | 125 | -39 | -34.5 | -16.5 | 4.0541 |
| Temporal_Inf_L | 92 | -49.5 | -46.5 | -6 | 3.5039 |
| left Hypothalamus | 287 | -7.5 | -6 | -4.5 | 3.9146 |
| Frontal_Mid_Orb_R | 116 | 39 | 46.5 | -7.5 | 3.7481 |
| Insula_L | 23 | -31.5 | 21 | 0 | 3.2054 |
| Cingulum_Ant_L | 127 | -10.5 | 42 | -1.5 | 3.669 |
| Occipital_Mid_R | 87 | 42 | -70.5 | 6 | 3.94 |
| Cingulum_Post_L | 21 | -12 | -42 | 7.5 | 3.4274 |
| Frontal_Inf_Tri_L | 173 | -39 | 28.5 | 10.5 | 4.0324 |
| Frontal_Sup_L | 31 | -21 | 52.5 | 9 | 3.4204 |
| Thalamus_R | 94 | 6 | -12 | 13.5 | 3.3368 |
| Occipital_Mid_R | 10 | 25.5 | -88.5 | 15 | 3.2774 |
| Frontal_Mid_L | 21 | -30 | 43.5 | 21 | 3.3944 |
| Frontal_Inf_Oper_L | 138 | -39 | 19.5 | 31.5 | 3.5911 |
| Occipital_Mid_R | 13 | 31.5 | -84 | 30 | 3.338 |
| Frontal_Inf_Oper_R | 45 | 40.5 | 16.5 | 34.5 | 3.5903 |
| Frontal_Mid_R | 88 | 33 | 4.5 | 49.5 | 4.0113 |
| Frontal_Sup_R | 10 | 27 | 24 | 52.5 | 3.2379 |

FDR<.05 corrected, Cluster size≥10; T represents the statistical value of the t-test for the regression coefficient β corresponding to the peak voxel.

| **Table S7 Correlation results between the relationship of VEM-GMV and age based on sliding window, Including sex differences in correlation coefficients (r).** | | | | | |
| --- | --- | --- | --- | --- | --- |
|  |  | r (FDRp) | | |  |
| Region | ROI in AAL90 | All Subjects | Female | Male | Fisher’s Z_diff(p) |
| Frontal | Precentral_L | 0.7(0.002) | 0.56(0.018) | 0.58(0.016) | 0.06(0.953) |
| Frontal | Precentral_R | 0.79(<0.001) | 0.71(<0.001) | 0.61(0.014) | 0.51(0.608) |
| Frontal | Frontal_Sup_L | 0.78(<0.001) | 0.24(0.413) | 0.73(0.009) | -1.01(0.313) |
| Frontal | Frontal_Sup_R | 0.68(0.003) | 0.71(<0.001) | 0.14(0.612) | 1.6(0.109) |
| Frontal | Frontal_Sup_Orb_L | 0.58(0.015) | 0.63(0.006) | 0.26(0.324) | 0.99(0.321) |
| Frontal | Frontal_Sup_Orb_R | 0.33(0.209) | 0.29(0.292) | 0.64(0.01) | -0.97(0.331) |
| Frontal | Frontal_Mid_L | 0.78(<0.001) | 0.62(0.007) | 0.72(0.009) | -0.32(0.751) |
| Frontal | Frontal_Mid_R | 0.73(<0.001) | 0.7(0.002) | 0.51(0.039) | 0.76(0.446) |
| Frontal | Frontal_Mid_Orb_R | 0.44(0.069) | 0.38(0.15) | 0.57(0.018) | -0.49(0.628) |
| Frontal | Frontal_Inf_Oper_L | 0.74(<0.001) | 0.7(0.002) | 0.45(0.066) | 0.94(0.345) |
| Frontal | Frontal_Inf_Oper_R | 0.71(0.002) | 0.45(0.068) | 0.61(0.014) | -0.27(0.789) |
| Frontal | Frontal_Inf_Tri_L | 0.83(0) | 0.71(<0.001) | 0.05(0.827) | 1.88(0.06) |
| Frontal | Frontal_Inf_Tri_R | 0.74(<0.001) | 0.77(<0.001) | 0.13(0.612) | 1.97(0.049) |
| Frontal | Frontal_Inf_Orb_L | 0.69(0.002) | 0.75(<0.001) | 0.42(0.092) | 1.21(0.226) |
| Frontal | Frontal_Inf_Orb_R | 0.68(0.003) | 0.65(0.005) | -0.15(0.591) | 1.81(0.07) |
| Frontal | Rolandic_Oper_L | 0.64(0.005) | 0.46(0.064) | 0.54(0.03) | -0.07(0.947) |
| Frontal | Rolandic_Oper_R | 0.58(0.015) | 0.52(0.029) | 0.6(0.016) | -0.22(0.829) |
| Frontal | Supp_Motor_Area_R | 0.49(0.041) | NaN | -0.5(0.04) | NaN |
| Frontal | Olfactory_L | 0.64(0.005) | 0.69(0.002) | 0.62(0.012) | 0.26(0.795) |
| Frontal | Olfactory_R | 0.5(0.035) | 0.48(0.046) | 0.58(0.016) | -0.3(0.764) |
| Frontal | Frontal_Sup_Medial_L | 0.82(0) | -0.05(0.881) | 0.5(0.04) | -0.14(0.891) |
| Frontal | Frontal_Sup_Medial_R | 0.65(0.005) | 0.55(0.02) | 0.51(0.039) | 0.22(0.825) |
| Frontal | Frontal_Mid_Orb_L | 0.76(<0.001) | 0.79(<0.001) | 0.47(0.056) | 1.36(0.173) |
| Frontal | Frontal_Mid_Orb_R | 0.25(0.373) | 0.78(<0.001) | -0.06(0.809) | 1.83(0.068) |
| Frontal | Rectus_L | 0.63(0.006) | 0.74(<0.001) | 0.12(0.647) | 1.73(0.084) |
| Frontal | Rectus_R | 0.33(0.204) | 0.6(0.009) | -0.64(0.01) | 1.84(0.066) |
| Frontal | Cingulum_Ant_L | 0.57(0.017) | 0.32(0.233) | 0.62(0.012) | -0.61(0.54) |
| Frontal | Cingulum_Mid_L | 0.01(0.989) | -0.01(0.973) | 0.59(0.016) | -1.32(0.186) |
| Frontal | Cingulum_Mid_R | 0.06(0.885) | -0.01(0.971) | 0.5(0.04) | -0.95(0.341) |
| Parietal | Cingulum_Post_L | 0.04(0.919) | 0.63(0.006) | 0.4(0.112) | 0.05(0.959) |
| Parietal | Cingulum_Post_R | -0.18(0.568) | 0.57(0.015) | NaN | NaN |
| Parietal | Postcentral_L | -0.17(0.568) | 0.59(0.011) | -0.67(0.009) | 1.13(0.258) |
| Parietal | Postcentral_R | 0.51(0.032) | 0.17(0.553) | 0.12(0.642) | 0.47(0.638) |
| Parietal | Parietal_Sup_L | -0.52(0.031) | 0.1(0.736) | -0.45(0.069) | -0.53(0.597) |
| Parietal | Parietal_Inf_L | -0.26(0.347) | 0.52(0.029) | -0.49(0.041) | 0.62(0.536) |
| Parietal | Parietal_Inf_R | 0.14(0.656) | 0.63(0.006) | 0.7(0.009) | -0.85(0.395) |
| Parietal | SupraMarginal_L | 0.38(0.136) | NaN | 0.48(0.048) | NaN |
| Parietal | SupraMarginal_R | -0.51(0.032) | 0.73(<0.001) | 0.65(0.009) | -1.5(0.133) |
| Parietal | Angular_L | -0.53(0.026) | 0.11(0.723) | 0.15(0.605) | -1.36(0.174) |
| Parietal | Angular_R | -0.31(0.236) | -0.24(0.414) | 0.66(0.009) | -2.09(0.037) |
| Parietal | Precuneus_L | 0.17(0.568) | 0.65(0.004) | 0.61(0.014) | -0.35(0.724) |
| Parietal | Precuneus_R | 0.51(0.032) | 0.55(0.02) | 0.58(0.016) | -0.14(0.887) |
| Parietal | Paracentral_Lobule_L | 0.58(0.015) | NaN | 0.5(0.04) | NaN |
| Occipital | Calcarine_L | 0.64(0.005) | 0.19(0.528) | NaN | NaN |
| Occipital | Cuneus_L | 0.64(0.005) | 0.36(0.166) | NaN | NaN |
| Occipital | Lingual_L | 0.75(<0.001) | 0.64(0.005) | 0.62(0.012) | 0.18(0.858) |
| Occipital | Lingual_R | 0.54(0.025) | 0.52(0.028) | 0.65(0.009) | -0.45(0.65) |
| Occipital | Occipital_Mid_L | -0.55(0.023) | NaN | 0.19(0.504) | NaN |
| Occipital | Occipital_Mid_R | -0.06(0.886) | -0.56(0.018) | 0.29(0.261) | -0.04(0.966) |
| Occipital | Occipital_Inf_L | -0.67(0.003) | 0.07(0.833) | -0.69(0.009) | -0.87(0.385) |
| Temporal | Hippocampus_L | 0.73(<0.001) | 0.74(<0.001) | 0.58(0.016) | 0.71(0.477) |
| Temporal | Hippocampus_R | 0.65(0.005) | 0.68(0.002) | 0.45(0.066) | 0.79(0.432) |
| Temporal | ParaHippocampal_L | 0.76(<0.001) | 0.75(<0.001) | 0.69(0.009) | 0.33(0.741) |
| Temporal | ParaHippocampal_R | 0.67(0.003) | 0.73(<0.001) | 0.65(0.009) | 0.34(0.737) |
| Temporal | Amygdala_L | 0.75(<0.001) | 0.74(<0.001) | 0.66(0.009) | 0.41(0.681) |
| Temporal | Amygdala_R | 0.59(0.012) | -0.02(0.949) | 0.65(0.009) | -0.88(0.38) |
| Temporal | Fusiform_L | 0.68(0.003) | 0.6(0.01) | 0.51(0.039) | 0.38(0.706) |
| Temporal | Fusiform_R | 0.57(0.017) | 0.07(0.833) | 0.68(0.009) | -1.03(0.305) |
| Temporal | Heschl_R | 0.45(0.063) | 0.56(0.017) | NaN | NaN |
| Temporal | Temporal_Sup_L | 0.47(0.054) | 0.28(0.318) | 0.53(0.033) | -0.43(0.666) |
| Temporal | Temporal_Sup_R | 0.63(0.007) | 0.57(0.015) | 0.68(0.009) | -0.39(0.699) |
| Temporal | Temporal_Pole_Sup_L | 0.68(0.003) | 0.74(<0.001) | 0.53(0.03) | 0.84(0.404) |
| Temporal | Temporal_Pole_Sup_R | 0.49(0.04) | 0.79(<0.001) | 0.59(0.016) | 0.73(0.466) |
| Temporal | Temporal_Mid_R | 0.00(0.992) | -0.78(<0.001) | -0.16(0.591) | 1.63(0.103) |
| Temporal | Temporal_Pole_Mid_L | 0.43(0.079) | 0.19(0.528) | 0.55(0.025) | -0.61(0.544) |
| Temporal | Temporal_Pole_Mid_R | 0.05(0.892) | 0.17(0.553) | 0.51(0.039) | -0.95(0.341) |
| Temporal | Temporal_Inf_L | 0.52(0.031) | 0.32(0.235) | 0.42(0.091) | -0.03(0.979) |
| Subcortical | Insula_L | 0.51(0.034) | 0.77(<0.001) | 0.39(0.116) | 1.22(0.221) |
| Subcortical | Insula_R | 0.7(0.002) | 0.71(<0.001) | 0.13(0.612) | 1.64(0.101) |
| Subcortical | Putamen_R | -0.52(0.032) | 0.17(0.553) | 0.02(0.921) | -1.09(0.277) |
| Subcortical | Pallidum_L | 0.19(0.532) | -0.09(0.791) | 0.52(0.037) | -0.83(0.408) |
| Subcortical | Pallidum_R | 0.02(0.952) | NaN | 0.53(0.03) | NaN |
| Subcortical | Thalamus_L | 0.73(<0.001) | 0.74(<0.001) | 0.55(0.026) | 0.81(0.416) |
| Subcortical | Thalamus_R | 0.73(<0.001) | 0.66(0.004) | 0.44(0.071) | 0.81(0.418) |

Only statistically significant results are presented, FDR<.05 corrected. Nan in Result of Fisher's z test indicates that the mean β within ROIs of all Windows with at least one of the two sex groups is nan.

| **Table S8 Correlation results between the relationship of SEM-GMV and age based on sliding window, Including sex differences in correlation coefficients (r).** | | | | | |
| --- | --- | --- | --- | --- | --- |
|  |  | r (FDRp) | | |  |
| Region | ROI in AAL90 | All Subjects | Female | Male | Fisher’s Z_diff(p) |
| Frontal | Precentral_L | 0.74(0) | 0.27(0.237) | 0.73(<0.001) | -1.02(0.308) |
| Frontal | Precentral_R | 0.69(<0.001) | 0.52(0.025) | 0.72(0.002) | -0.65(0.516) |
| Frontal | Frontal_Sup_L | 0.78(0) | 0.59(0.01) | 0.67(0.003) | -0.16(0.876) |
| Frontal | Frontal_Sup_R | 0.87(0) | 0.63(0.005) | 0.48(0.039) | 0.72(0.474) |
| Frontal | Frontal_Sup_Orb_L | 0.76(0) | 0.72(<0.001) | 0.59(0.009) | 0.61(0.544) |
| Frontal | Frontal_Sup_Orb_R | 0.76(0) | 0.32(0.181) | 0.74(<0.001) | -1.01(0.312) |
| Frontal | Frontal_Mid_L | 0.76(0) | 0.8(0) | 0.71(0.002) | 0.52(0.601) |
| Frontal | Frontal_Mid_R | 0.75(0) | 0.7(<0.001) | 0.59(0.009) | 0.51(0.609) |
| Frontal | Frontal_Mid_Orb_L | 0.71(<0.001) | 0.25(0.271) | 0.67(0.003) | -0.76(0.446) |
| Frontal | Frontal_Mid_Orb_R | 0.74(0) | 0.55(0.017) | 0.74(<0.001) | -0.65(0.516) |
| Frontal | Frontal_Inf_Oper_L | 0.74(0) | 0.54(0.02) | 0.67(0.003) | -0.32(0.753) |
| Frontal | Frontal_Inf_Oper_R | 0.69(<0.001) | 0.5(0.032) | 0.37(0.112) | 0.53(0.599) |
| Frontal | Frontal_Inf_Tri_L | 0.63(0.004) | 0.61(0.007) | 0.52(0.024) | 0.33(0.741) |
| Frontal | Frontal_Inf_Tri_R | 0.61(0.004) | NaN | 0.53(0.023) | NaN |
| Frontal | Frontal_Inf_Orb_L | 0.69(<0.001) | 0.42(0.075) | 0.4(0.09) | 0.31(0.755) |
| Frontal | Frontal_Inf_Orb_R | 0.63(0.004) | 0.67(0.002) | 0.62(0.006) | 0.18(0.861) |
| Frontal | Rolandic_Oper_L | 0.76(0) | 0.41(0.084) | 0.73(<0.001) | -0.84(0.401) |
| Frontal | Rolandic_Oper_R | 0.73(0) | NaN | 0.45(0.059) | NaN |
| Frontal | Supp_Motor_Area_L | 0.71(<0.001) | 0.68(0.002) | NaN | NaN |
| Frontal | Supp_Motor_Area_R | 0.63(0.003) | 0.51(0.028) | -0.02(0.923) | 1.21(0.225) |
| Frontal | Olfactory_L | 0.81(0) | 0.83(0) | 0.69(0.002) | 0.88(0.376) |
| Frontal | Olfactory_R | 0.73(0) | 0.86(0) | 0.65(0.004) | 1.27(0.206) |
| Frontal | Frontal_Sup_Medial_L | 0.85(0) | NaN | 0.78(<0.001) | NaN |
| Frontal | Frontal_Sup_Medial_R | 0.62(0.004) | NaN | 0.4(0.088) | NaN |
| Frontal | Frontal_Mid_Orb_L | 0.74(0) | 0.8(0) | 0.66(0.003) | 0.75(0.456) |
| Frontal | Frontal_Mid_Orb_R | 0.72(<0.001) | NaN | 0.26(0.282) | NaN |
| Frontal | Rectus_L | 0.8(0) | 0.71(<0.001) | 0.66(0.003) | 0.32(0.752) |
| Frontal | Rectus_R | 0.67(0.002) | 0.28(0.237) | 0.62(0.006) | -0.56(0.576) |
| Frontal | Cingulum_Ant_L | 0.75(0) | 0.45(0.056) | 0.7(0.002) | -0.63(0.531) |
| Frontal | Cingulum_Ant_R | 0.22(0.337) | 0.59(0.01) | -0.13(0.614) | 1.12(0.261) |
| Frontal | Cingulum_Mid_L | 0.72(<0.001) | NaN | NaN | NaN |
| Frontal | Cingulum_Mid_R | 0.72(<0.001) | 0.51(0.029) | NaN | NaN |
| Parietal | Cingulum_Post_L | 0.53(0.015) | 0.34(0.155) | NaN | NaN |
| Parietal | Cingulum_Post_R | 0.52(0.018) | NaN | NaN | NaN |
| Parietal | Postcentral_L | 0.67(<0.001) | NaN | 0.46(0.051) | NaN |
| Parietal | Postcentral_R | 0.44(0.049) | NaN | 0.12(0.617) | NaN |
| Parietal | Parietal_Sup_L | 0.54(0.013) | NaN | 0.6(0.009) | NaN |
| Parietal | Parietal_Sup_R | 0.78(0) | NaN | 0.73(<0.001) | NaN |
| Parietal | Parietal_Inf_L | 0.7(<0.001) | NaN | 0.56(0.015) | NaN |
| Parietal | Parietal_Inf_R | 0.72(<0.001) | NaN | 0.47(0.048) | NaN |
| Parietal | SupraMarginal_L | 0.52(0.017) | NaN | NaN | NaN |
| Parietal | Angular_L | 0.72(<0.001) | NaN | 0.37(0.115) | NaN |
| Parietal | Angular_R | 0.73(0) | NaN | 0.62(0.006) | NaN |
| Parietal | Precuneus_L | 0.5(0.023) | 0.37(0.128) | 0.55(0.017) | -0.37(0.712) |
| Parietal | Precuneus_R | 0.71(<0.001) | NaN | 0.75(<0.001) | NaN |
| Parietal | Paracentral_Lobule_L | 0.53(0.014) | NaN | NaN | NaN |
| Parietal | Paracentral_Lobule_R | 0.59(0.006) | NaN | 0.59(0.009) | NaN |
| Occipital | Calcarine_R | 0.18(0.443) | NaN | 0.58(0.011) | NaN |
| Occipital | Cuneus_L | 0.7(<0.001) | NaN | 0.68(0.003) | NaN |
| Occipital | Cuneus_R | 0.61(0.004) | NaN | 0.44(0.06) | NaN |
| Occipital | Lingual_L | 0.61(0.004) | NaN | 0.64(0.004) | NaN |
| Occipital | Lingual_R | 0.47(0.031) | NaN | 0.74(<0.001) | NaN |
| Occipital | Occipital_Sup_L | 0.76(0) | NaN | 0.68(0.003) | NaN |
| Occipital | Occipital_Sup_R | 0.59(0.006) | -0.12(0.605) | NaN | NaN |
| Occipital | Occipital_Mid_L | 0.67(0.002) | NaN | 0.28(0.254) | NaN |
| Occipital | Occipital_Mid_R | 0.57(0.009) | 0.6(0.009) | 0.73(<0.001) | -0.62(0.537) |
| Occipital | Occipital_Inf_L | 0.82(0) | NaN | 0.24(0.316) | NaN |
| Occipital | Occipital_Inf_R | 0.59(0.006) | NaN | -0.51(0.026) | NaN |
| Temporal | Hippocampus_L | 0.73(0) | 0.76(0) | 0.4(0.088) | 1.35(0.178) |
| Temporal | Hippocampus_R | 0.73(0) | 0.84(0) | 0.42(0.075) | 1.84(0.066) |
| Temporal | ParaHippocampal_L | 0.69(<0.001) | 0.57(0.013) | 0.63(0.005) | -0.11(0.909) |
| Temporal | ParaHippocampal_R | 0.65(0.002) | 0.75(0) | 0.52(0.025) | 0.89(0.373) |
| Temporal | Amygdala_L | 0.76(0) | 0.54(0.02) | 0.64(0.004) | -0.17(0.867) |
| Temporal | Amygdala_R | 0.83(0) | 0.75(0) | 0.59(0.009) | 0.81(0.419) |
| Temporal | Fusiform_L | 0.59(0.006) | 0.3(0.199) | 0.58(0.011) | -0.46(0.643) |
| Temporal | Fusiform_R | 0.64(0.003) | 0.7(<0.001) | 0.6(0.008) | 0.38(0.706) |
| Temporal | Heschl_L | 0.77(0) | 0.44(0.059) | 0.68(0.003) | -0.53(0.596) |
| Temporal | Heschl_R | 0.61(0.004) | 0.46(0.053) | NaN | NaN |
| Temporal | Temporal_Sup_L | 0.69(<0.001) | 0.32(0.182) | 0.7(0.002) | -0.86(0.392) |
| Temporal | Temporal_Sup_R | 0.75(0) | 0.34(0.161) | 0.64(0.004) | -0.51(0.611) |
| Temporal | Temporal_Pole_Sup_L | 0.54(0.013) | 0.75(0) | 0.37(0.112) | 1.19(0.232) |
| Temporal | Temporal_Pole_Sup_R | 0.58(0.007) | 0.76(0) | 0.08(0.762) | 1.85(0.064) |
| Temporal | Temporal_Mid_L | 0.82(0) | 0.7(<0.001) | 0.68(0.003) | 0.2(0.842) |
| Temporal | Temporal_Mid_R | 0.73(0) | 0.28(0.237) | 0.42(0.076) | 0.13(0.899) |
| Temporal | Temporal_Pole_Mid_L | 0.56(0.01) | 0.67(0.002) | 0.27(0.254) | 1.09(0.276) |
| Temporal | Temporal_Pole_Mid_R | 0.56(0.01) | 0.58(0.012) | 0.3(0.208) | 0.75(0.455) |
| Temporal | Temporal_Inf_L | 0.83(0) | 0.45(0.056) | 0.57(0.012) | -0.02(0.984) |
| Temporal | Temporal_Inf_R | 0.71(<0.001) | 0.77(0) | 0.15(0.546) | 1.91(0.056) |
| Subcortical | Insula_L | 0.76(0) | 0.78(0) | 0.54(0.02) | 1.09(0.276) |
| Subcortical | Insula_R | 0.8(0) | 0.51(0.028) | 0.65(0.004) | -0.24(0.809) |
| Subcortical | Caudate_L | 0.78(0) | 0.83(0) | 0.04(0.887) | 2.56(0.011) |
| Subcortical | Caudate_R | 0.68(<0.001) | NaN | 0.67(0.003) | NaN |
| Subcortical | Putamen_L | 0.71(<0.001) | 0.82(0) | 0.68(0.003) | 0.77(0.442) |
| Subcortical | Putamen_R | 0.7(<0.001) | NaN | 0.7(0.002) | NaN |
| Subcortical | Pallidum_L | 0.62(0.004) | NaN | 0.39(0.1) | NaN |
| Subcortical | Pallidum_R | 0.51(0.02) | NaN | 0.5(0.029) | NaN |
| Subcortical | Thalamus_L | 0.73(0) | 0.49(0.035) | 0.74(<0.001) | -0.79(0.432) |
| Subcortical | Thalamus_R | 0.83(0) | 0.71(<0.001) | 0.73(<0.001) | -0.01(0.996) |

Only statistically significant results are presented, FDR<.05 corrected. Nan in Result of Fisher's z test indicates that the mean β within ROIs of all Windows with at least one of the two sex groups is nan.

| Table S9 Partial correlation between GMV and age based on AAL90 | | | |
| --- | --- | --- | --- |
| ROI in AAL90 | β  male | β  female | Significance of  differences of β |
| Precentral_L | -0.00131 | -0.00126 | <.05 |
| Precentral_R | -0.00133 | -0.00127 | <.05 |
| Frontal_Sup_L | -0.00104 | -0.001 | 0.888 |
| Frontal_Sup_R | -0.00095 | -0.00092 | 0.715 |
| Frontal_Sup_Orb_L | -0.00171 | -0.00165 | 0.182 |
| Frontal_Sup_Orb_R | -0.00156 | -0.00151 | 0.412 |
| Frontal_Mid_L | -0.00129 | -0.00124 | 0.347 |
| Frontal_Mid_R | -0.00128 | -0.00123 | 0.133 |
| Frontal_Mid_Orb_L | -0.00159 | -0.00154 | 0.157 |
| Frontal_Mid_Orb_R | -0.0013 | -0.00124 | 0.715 |
| Frontal_Inf_Oper_L | -0.00142 | -0.00137 | 0.056 |
| Frontal_Inf_Oper_R | -0.0012 | -0.00115 | <.01 |
| Frontal_Inf_Tri_L | -0.00118 | -0.00113 | 0.169 |
| Frontal_Inf_Tri_R | -0.001 | -0.00096 | <.05 |
| Frontal_Inf_Orb_L | -0.00167 | -0.00161 | 0.791 |
| Frontal_Inf_Orb_R | -0.00147 | -0.00142 | 0.714 |
| Rolandic_Oper_L | -0.00171 | -0.00164 | 0.215 |
| Rolandic_Oper_R | -0.00166 | -0.0016 | 0.243 |
| Supp_Motor_Area_L | -0.00113 | -0.00109 | 0.167 |
| Supp_Motor_Area_R | -0.00119 | -0.00116 | 0.638 |
| Olfactory_L | -0.00287 | -0.00276 | 0.18 |
| Olfactory_R | -0.00236 | -0.00227 | 0.653 |
| Frontal_Sup_Medial_L | -0.00092 | -0.00089 | 0.314 |
| Frontal_Sup_Medial_R | -0.00078 | -0.00075 | 0.576 |
| Frontal_Mid_Orb_L | -0.00175 | -0.00169 | 0.732 |
| Frontal_Mid_Orb_R | -0.00203 | -0.00196 | 0.523 |
| Rectus_L | -0.00198 | -0.00192 | 0.575 |
| Rectus_R | -0.00208 | -0.00202 | 0.458 |
| Insula_L | -0.00146 | -0.00141 | 0.304 |
| Insula_R | -0.00196 | -0.00191 | 0.421 |
| Cingulum_Ant_L | -0.00235 | -0.00226 | 0.828 |
| Cingulum_Ant_R | -0.00192 | -0.00184 | 0.551 |
| Cingulum_Mid_L | -0.00182 | -0.00175 | 0.212 |
| Cingulum_Mid_R | -0.0016 | -0.00155 | 0.159 |
| Cingulum_Post_L | -0.00184 | -0.00182 | 0.301 |
| Cingulum_Post_R | -0.00112 | -0.00108 | 0.129 |
| Hippocampus_L | -0.00354 | -0.00343 | 0.963 |
| Hippocampus_R | -0.00339 | -0.00329 | 0.961 |
| ParaHippocampal_L | -0.00262 | -0.00254 | 0.568 |
| ParaHippocampal_R | -0.00337 | -0.00328 | 0.519 |
| Amygdala_L | -0.00366 | -0.00356 | 0.809 |
| Amygdala_R | -0.00313 | -0.00307 | 0.799 |
| Calcarine_L | -0.00229 | -0.00223 | 0.244 |
| Calcarine_R | -0.00212 | -0.00207 | 0.483 |
| Cuneus_L | -0.00156 | -0.00152 | <.01 |
| Cuneus_R | -0.00162 | -0.00159 | 0.303 |
| Lingual_L | -0.00182 | -0.00177 | 0.142 |
| Lingual_R | -0.00186 | -0.00182 | 0.878 |
| Occipital_Sup_L | -0.00149 | -0.00144 | 0.141 |
| Occipital_Sup_R | -0.00149 | -0.00144 | 0.403 |
| Occipital_Mid_L | -0.00148 | -0.00144 | <.01 |
| Occipital_Mid_R | -0.00153 | -0.00149 | 0.236 |
| Occipital_Inf_L | -0.00184 | -0.00181 | 0.848 |
| Occipital_Inf_R | -0.00138 | -0.00135 | 0.614 |
| Fusiform_L | -0.00289 | -0.00281 | 0.393 |
| Fusiform_R | -0.00284 | -0.00276 | 0.287 |
| Postcentral_L | -0.00121 | -0.00115 | <.05 |
| Postcentral_R | -0.00084 | -0.00081 | <.01 |
| Parietal_Sup_L | -0.00102 | -0.00097 | 0.576 |
| Parietal_Sup_R | -0.00052 | -0.0005 | 0.064 |
| Parietal_Inf_L | -0.00118 | -0.00115 | <.05 |
| Parietal_Inf_R | -0.00131 | -0.00128 | 0.245 |
| SupraMarginal_L | -0.00082 | -0.00079 | <.05 |
| SupraMarginal_R | -0.00136 | -0.00133 | <.05 |
| Angular_L | -0.00111 | -0.00108 | <.01 |
| Angular_R | -0.00142 | -0.00138 | 0.167 |
| Precuneus_L | -0.0011 | -0.00108 | 0.913 |
| Precuneus_R | -0.00121 | -0.00119 | 0.401 |
| Paracentral_Lobule_L | -0.00113 | -0.00108 | 0.119 |
| Paracentral_Lobule_R | -0.00124 | -0.00119 | 0.416 |
| Caudate_L | -0.00071 | -0.00068 | 0.983 |
| Caudate_R | -0.00013 | -0.00013 | 0.954 |
| Putamen_L | -0.00023 | -0.00023 | 0.147 |
| Putamen_R | 1.25E-05 | -2.9E-06 | 0.097 |
| Pallidum_L | 0.000914 | 0.0009 | 0.834 |
| Pallidum_R | 0.001281 | 0.001231 | 0.463 |
| Thalamus_L | -0.00199 | -0.00194 | 0.647 |
| Thalamus_R | -0.00161 | -0.00158 | 0.416 |
| Heschl_L | -0.00272 | -0.00262 | 0.992 |
| Heschl_R | -0.00315 | -0.00304 | 0.887 |
| Temporal_Sup_L | -0.00153 | -0.00148 | <.05 |
| Temporal_Sup_R | -0.00158 | -0.00152 | 0.089 |
| Temporal_Pole_Sup_L | -0.00198 | -0.00192 | 0.944 |
| Temporal_Pole_Sup_R | -0.00191 | -0.00184 | 0.76 |
| Temporal_Mid_L | -0.0021 | -0.00204 | <.05 |
| Temporal_Mid_R | -0.00194 | -0.00189 | 0.373 |
| Temporal_Pole_Mid_L | -0.00233 | -0.00224 | 0.157 |
| Temporal_Pole_Mid_R | -0.00225 | -0.00217 | 0.892 |
| Temporal_Inf_L | -0.00241 | -0.00235 | 0.134 |
| Temporal_Inf_R | -0.00213 | -0.00207 | 0.371 |
| AT system | -0.000413 | 0.000003 | 0.907 |
| PM system | -0.000177 | -0.000008 | 0.446 |


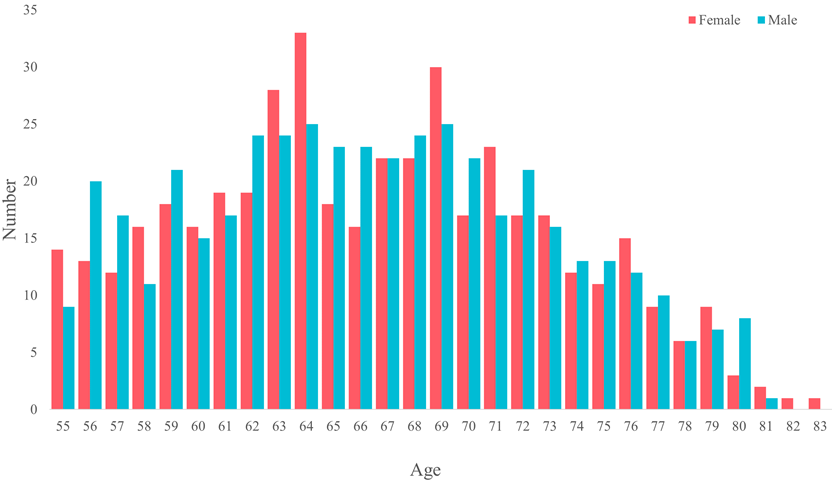


Fig S10 The age distribution of the sample used in the study.


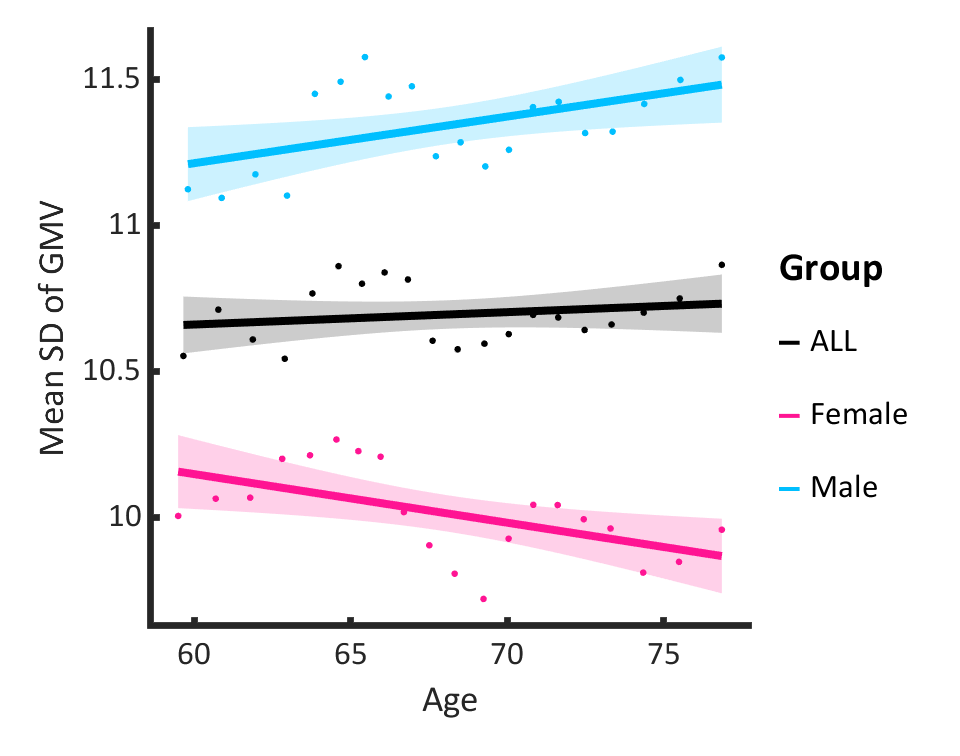


**Fig S11 The relationship between voxel-based GMV standard deviation for each window and age.**

We separately calculated the standard deviation of each voxel for all subjects, male subjects, and female subjects within each window. Specifically, assuming the matrix is *M_ijk_*, we iterated through each voxel and calculated the standard deviation for the corresponding voxel across all participants within the window, representing the variability in the respective region. The average standard deviation for the entire brain was then computed, serving as the measure of the mean variability within that window. Pearson correlation analyses were conducted between the average variability across all windows and age. The results revealed a non-significant correlation for ALL subjects (r = 0.206, p = 0.384), a significant positive correlation for the Male group (r = 0.517, p = 0.0196), and a significant negative correlation for the Female group (r = -0.546, p = 0.0129). An independent samples t-test was conducted to compare the average variability levels between different sexes The results revealed a significant difference, with males showing higher variability than females (t = 28.40, p < 0.001).

**The influence of varying overlaps in sliding windows on the correlation between GMV-EM association and age**


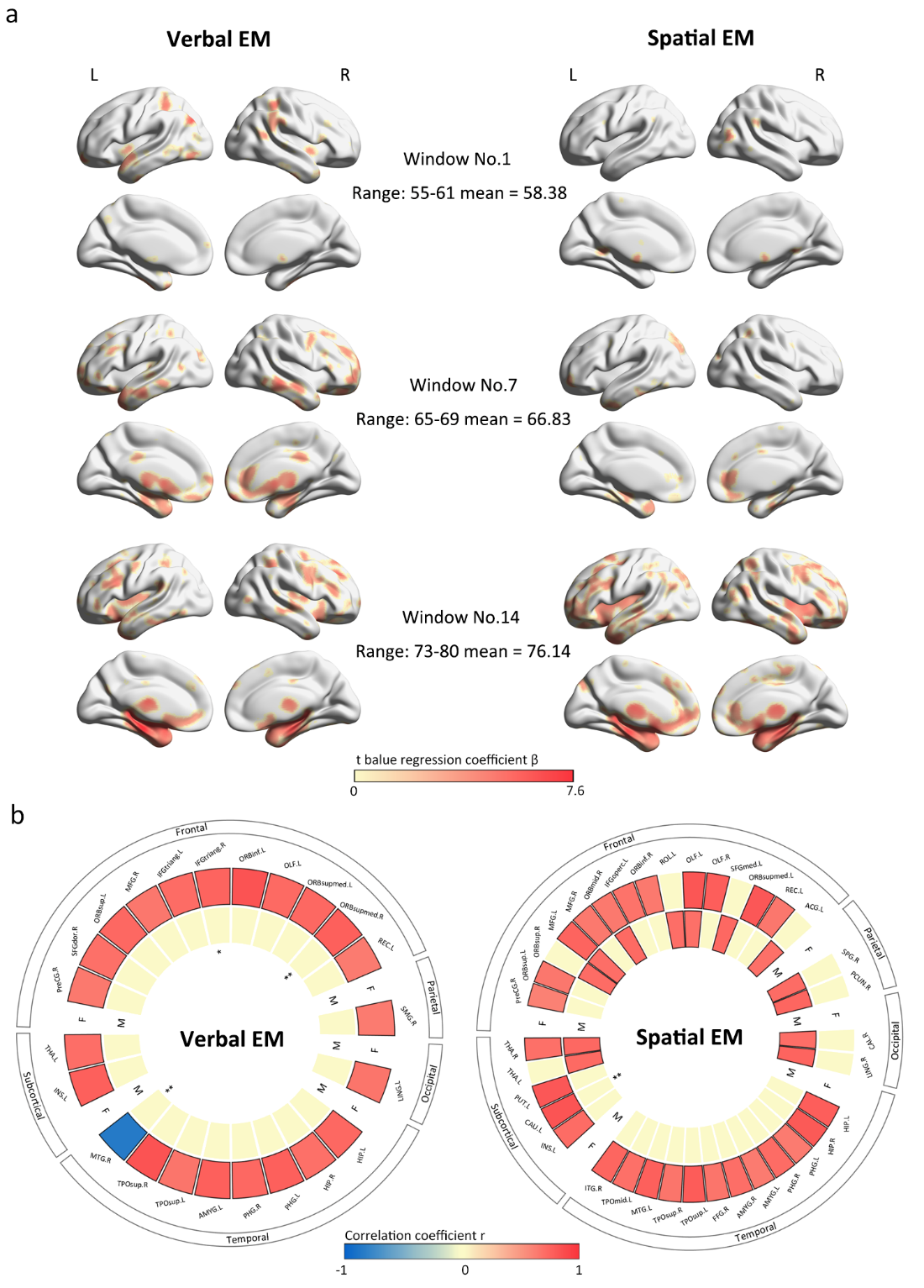


**Fig S12 Age differences in the correlation between GMV and two types of EM (overlap=70%)**


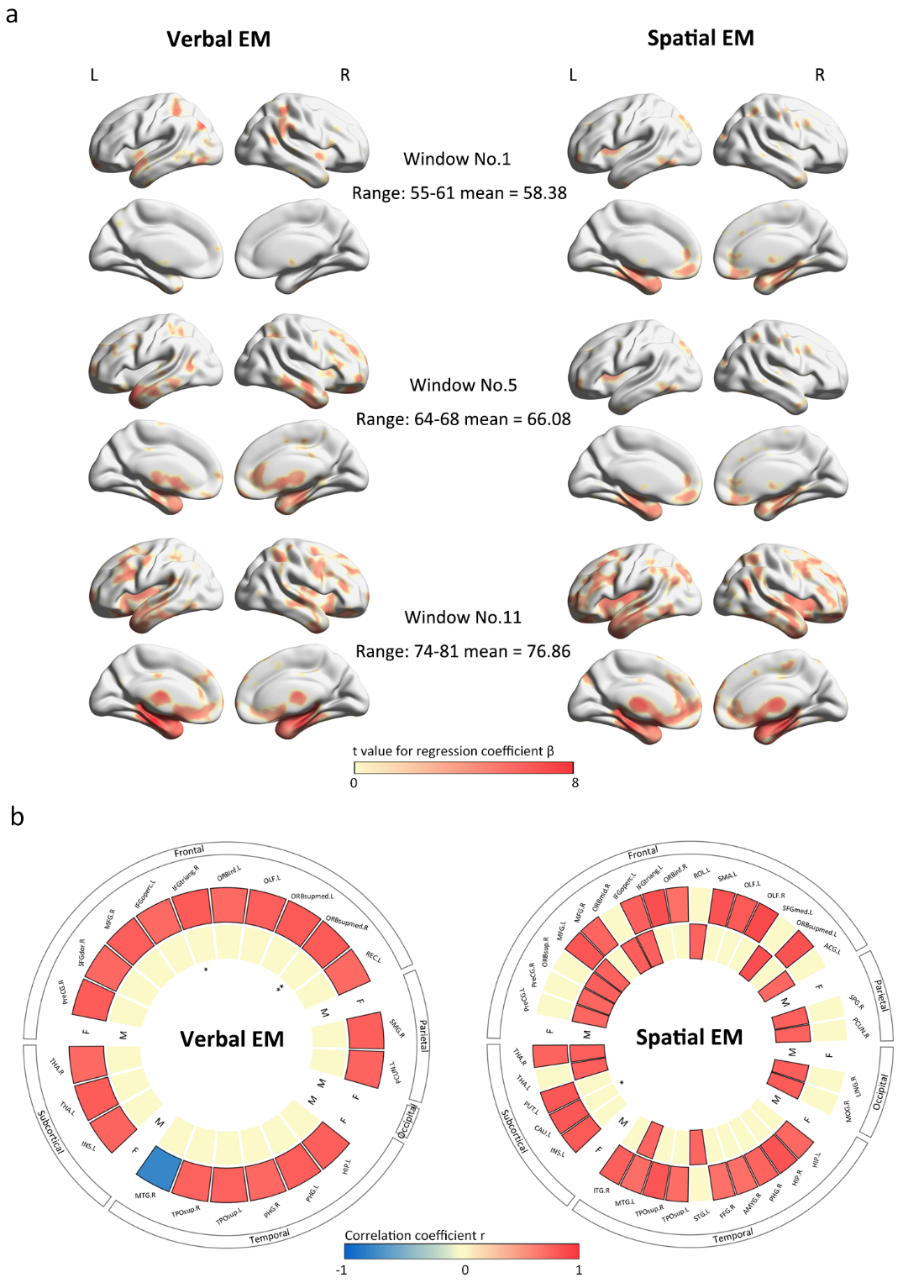


**Fig S13 Age differences in the correlation between GMV and two types of EM (overlap=60%)**

In addition to the 80% overlap reported in the main text, we also examined the results of correlation analyses for the relationship between GMV-EM and age with two different overlaps, 60% (S12) and 70% (S13). We observed that, under different overlap settings, smaller overlap values led to a reduction in the number of windows delineated along the entire age axis (21 windows with 80% overlap vs. 14 windows with 70% overlap vs. 11 windows with 60% overlap). Furthermore, the decrease in the number of windows posed a challenge to establishing a significant correlation between GMV-EM and age. However, the correlation between GMV-EM and age maintained a stable positive trend across all three overlap scenarios. To allocate more windows within the constraints of a limited dataset, we opted for an 80% overlap, providing a more comprehensive depiction of the evolving relationship between GMV-EM correlation and age.
